# Causal relationships between serum matrix metalloproteinases and estrogen receptor-negative breast cancer: a bidirectional mendelian randomization study

**DOI:** 10.1038/s41598-023-34200-0

**Published:** 2023-05-15

**Authors:** Zijun Zhao, Qing Cao, Ming Zhu, Chaonan Wang, Xin Lu

**Affiliations:** Department of Surgery, Peking Union Medical College Hospital, Chinese Academy of Medical Sciences and Peking Union Medical College, 1 Shuaifuyuan, Wangfujing, Beijing, 100730 China

**Keywords:** Cancer, Genetics, Biomarkers

## Abstract

To better clarify the causal effects between matrix metalloproteinases (MMPs) and estrogen-receptor (ER)-negative breast cancer (BC), we investigated the bidirectional causal relationship between MMPs and ER-negative BC by mendelian randomization (MR) analysis. Summary statistic data of five MMPs were extracted from European participants in 13 cohorts. Data of ER-negative BC collected from one of genome-wide association studies of European ancestry was used as experimental datasets and another four ER-negative BC datasets were used as validation sets. Inverse variance weighted method was used for main MR analysis and sensitivity analysis was also conducted. Serum level of MMP-1 has negative effect on ER-negative BC (odds ratio = 0.92, *P* = 0.0008) but the latter one was not the cause of the former one, which was supported by validation sets. No bidirectional causal effect was detected between the other four types of MMPs and ER-negative BC (*P* > 0.05). Sensitivity analysis indicated robustness of the above results without remarkable bias. To conclude, serum MMP-1 may be a protective factor against ER-negative BC. No reciprocal causality was found between the other kinds of MMPs and ER-negative BC. MMP-1 was indicated as a biomarker for risk of ER-negative BC.

## Introduction

According to the latest version of global cancer statistic GLOBOCAN published in 2020, female breast cancer (BC) was the most common solid malignancy, with approximately 2.3 million newly diagnosed cases (11.7%). It took the fourth place (6.9%) regarding cause of cancer-specific death of female patients globally^1^. Conventionally, BC includes four main subtypes. Luminal A, Luminal B, human epidermal growth factor receptor 2 (HER-2) positive, and triple-negative BC (TNBC)^2^. The classification is mainly dependent on status of estrogen receptor (ER) and HER-2, indicating different clinical managements to different subtypes of BC^3^. ER is an essential predictor for response of endocrine therapy and prognosis of BC patients. Regarding ER-positive BC, endocrine therapy could largely reduce recurrence and mortality rate^4^. Patients with ER-negative BC has a relatively more aggressive biological trait and a worse prognosis than ER-positive BC after endocrine therapy^5^. HER-2 positive BC and TNBC are two special types of ER-negative BC. The former one accounts for around 15% of BC patients and possesses aggressive clinical features and results in a poor prognosis until the appearance of anti-HER-2 monoclonal antibody (trastuzumab and pertuzumab, etc.), which lifts response rate and improvs survival^6–9^. Resistance and recurrence, however, usually occur in HER-2 positive tumor, especially for advanced and metastatic one^10,11^. Different from the other three subtypes, TNBC lacks of expression of neither ER or HER-2, rendering it the most aggressive and refractory BC subtype especially in younger patients^12,13^. Commonly-used endocrine treatment (tamoxifen and aromatase inhibitors) and targeting anti-HER-2 therapy trastuzumab are ineffective in patients with TNBC^14^. Thus, it is indispensable to find some other targets to improve the treatment response of ER-negative BC.

Matrix metalloproteinases (MMPs) are a family of zinc-dependent endopeptidases, a subgroup of metzincin superfamily. In 1962, MMPs were first reported by Charles Lapiere and Jerome Gross in tadpole undergoing metamorphosis^15,16^. Totally, MMP include 26 zinc-dependent endopeptidases, among which 23 genes of MMP have been identified in *homo sapiens*^16,17^. Based on molecular structure and substrate specificity, MMPs in vertebrates can be classified in 6 groups: collagenases (MMP-1, -8, -13, and -18), gelatinases (MMP-2 and -9), matrilysins (MMP-7 and -26), stromelysins (MMP-3, -10, and -11), and transmembrane MMPs (MT-MMPs, MMP-14, -15, -16, -17, -24, and -25), and other unspecific types (MMP-12, -19, -20, -21, -22, -23, -27, and -28)^16^. Functionally, MMPs are involved in both physiological (degradation of extracellular matrix, embryonic growth, reproduction, etc.)^18^ and pathological process (aneurysms, atherosclerosis, arthritis, fibrosis, nephritis, tissue ulcers, and cancer)^19,20^. In tumor, specifically, MMPs degraded proteins in basement membrane and extracellular matrix, eliminating barriers against cancer cell invasion, facilitating the process of cancer progression and metastasis^21^. Previous studies have found relationships between single nucleotide polymorphisms (SNP) of MMPs genes and solid malignancies including lung cancer, esophageal cancer, head and neck cancer, colorectal cancer, and BC^22–24^.

Previous systemic review and meta-analysis suggested that several types of MMPs were associated with BC^25^. Huang study found that SNP of MMP-9 rs3918242 was remarkably relevant with incidence of BC among the overall population and Asian population^25^, which was supported by Xu study, indicating that one of MMP-9 polymorphisms, rs3918242, may be a risk factor of BC^26^. Another meta-analysis published by Han et.al corroborated this conclusion, indicating that MMP-9–1562 C/T polymorphism was a risk factor of BC, especially in European population whereas MMP2 polymorphism MMP-2–1306 C/T polymorphism was a protecting factor for BC in Asian population^27^. In a study from Ou et.al, however, no correlation was found between MMP-2–1306 C/T and risk of BC^28^. Ren and Song study demonstrated that MMP-9 overexpression was associated with a poorer overall survival and Ren study did not find significant impact of MMP-2 on prognosis of BC patients^29,30^. Different from the above research, Chen er.al reported that MMP-2 expression was significantly associated with a poor survival and risk of lymph node metastasis^31^. For SNP of MMP-1, the results were also controversial. Sui study showed that SNP of MMP-1 rs1799750 was related to a reduction of risk of BC in both the overall population and Asian group^32^. On the contrary, MMP-1 1G/2G genotype and MMP-1 2G/2G genotype were significantly associated with metastasis of BC^33^.

The above studies not only failed to draw consistent conclusion, but also did not confirm a causal relationship between MMPs and risk of BC in that all meta-analysis were based on observational (case–control) studies. Moreover, few study focused on the association of MMPs specifically with ER-negative BC. The traditional epidemiological approach is vulnerable to confounders and reverse causality, causing conflicting evidence^34,35^. To neutralize the adverse effect of confounders and reverse causality, we used the Mendelian Randomization (MR) method^36^. MR uses genetic variants (SNP) strongly associated with certain type of exposure as instrumental variables (IVs) to predict causality of the given exposure on an outcome of interest^37–39^. Genetic variants are randomly assorted, hardly modified during meiosis and they are also unrelated with other confounders (socioeconomical and environmental factors). Thus, different outcomes between populations with and without these IVs can be attributed to this exposure^39^. The results of MR estimation are reliable to reflect life-long exposure and reduce the impact of confounding factors and reverse causation^39–42^. As an extension of basic MR analysis, bidirectional MR can further validate whether two phenotypes can interact as reciprocal causality^43^. Herein, we aimed to explore the bidirectional causal relationship between several types of MMPs and ER-negative BC via two-sample MR analysis to find new targets for BC treatment.

## Results

### Causal effect of serum MMP levels on ER-negative BC

Ten types of MMPs (MMP-1, MMP-2, MMP-3, MMP-7, MMP-9, MMP-10, MMP-12, MMP-14, MMP-16, MMP-17) were available in the open GWAS summary datasets. After excluded MMPs with less than five significant associated SNPs (*P* < 5 × 10^−8^), five SNPs (MMP-1, MMP-3, MMP-7, MMP-10, MMP-12) were selected for following analysis. After extracting IVs of the five MMPs, we input these IVs into Phenoscanner database to remove SNPs associated with confounding factors. No confounder-related SNPs had been found for MMP-1, MMP-7, and MMP-12 whereas each of two SNPs (rs17360661, rs2267373 for MMP-3; rs3129886 and rs601338 for MMP-10) were found to be associated with BCs (cause of death; body weight; long-standing illness; alcohol intake). The number of ultimately selected LD-independent SNPs for data harmonization were 17, seven, nine, nine, and 15 for MMP-1, MMP-3, MMP-7, MMP-10, and MMP-12, respectively. F-statistic for SNPs of all the five MMPs were greater than the threshold of 10, suggesting strong IVs, which reducing bias of IVs estimates (Supplementary Tables S1, S2, S3, S4, S5). During harmonization, two, three, and one palindromic SNP(s) significantly associated with MMP-1, MMP-3, and MMP-12 were removed for following MR analysis. No palindromic SNPs were removed for MMP-7 and MMP-10.

The first dataset of BC (GWAS ID: ieu-a-1128) was used as experimental set to explore the causal effect of MMPs on ER-negative BC. Genetically elevated serum MMP-1 level were causally associated with a low risk of ER-negative BC (OR = 0.92, 95% confidence interval [CI]: 0.88–0.97, *P* = 0.0008), which was validated in the other three datasets as suggestive associations (ieu-a-1135: OR = 0.93, 95% CI: 0.87–0.99, *P* = 0.03; ieu-a-1136: OR = 0.92, 95% CI: 0.86–1.00, *P* = 0.049; ieu-a-1166: OR = 0.92, 95% CI: 0.85–1.00, *P* = 0.047) (Table 1). Genetically elevated serum MMP-3 level were not causally associated with risk of ER-negative BC (OR = 1.01, 95%CI: 0.93–1.09, *P* = 0.88), which was supported by the results of the other four datasets (ieu-a-1135: OR = 0.99, 95%CI: 0.91–1.08; *P* = 0.88; ieu-a-1136: OR = 0.99, 95% CI: 0.86–1.14, *P* = 0.884; ieu-a-1137: OR = 1.08, 95%CI: 0.97–1.20, *P* = 0.17; ieu-a-1166: OR = 1.00, 95%CI: 0.89–1.12, *P* = 1.00)(Table 2). Genetically elevated serum MMP-7 level were not causally associated with risk of ER-negative BC (OR = 1.05, 95%CI: 0.97–1.14, *P* = 0.24), which was supported by the results of the other four datasets (ieu-a-1135: OR = 0.98, 95%CI: 0.89–1.07; *P* = 0.60; ieu-a-1136: OR = 1.10, 95% CI: 0.96–1.26, *P* = 0.19; ieu-a-1137: OR = 1.22, 95%CI: 0.99–1.50, *P* = 0.06; ieu-a-1166: OR = 1.08, 95%CI: 0.94–1.25, *P* = 0.28) (Table 3). Genetically elevated serum MMP-10 level were not causally associated with risk of ER-negative BC (OR = 1.00, 95%CI: 0.95–1.06, *P* = 0.86), which was supported by the results of the other four datasets (ieu-a-1135: OR = 0.99, 95%CI: 0.93–1.06; *P* = 0.86; ieu-a-1136: OR = 1.00, 95% CI: 0.89–1.13, *P* = 1.00; ieu-a-1137: OR = 1.05, 95%CI: 0.93–1.19, *P* = 0.43; ieu-a-1166: OR = 1.00, 95%CI: 0.89–1.13, *P* = 0.98) (Table 4). Genetically elevated serum MMP-12 level were not causally associated with risk of ER-negative BC (OR = 1.02, 95%CI: 0.96–1.07, *P* = 0.56), which was supported by the results of the other three datasets (ieu-a-1136: OR = 0.98, 95% CI: 0.91–1.05, *P* = 0.59; ieu-a-1137: OR = 0.95, 95%CI: 0.85–1.06, *P* = 0.35; ieu-a-1166: OR = 0.99, 95%CI: 0.92–1.07, *P* = 0.86) except GWAS ieu-a-1135 (OR = 1.07, 95%CI: 1.00–1.13, *P* = 0.04) (Table 5). All the results above were calculated by IVW method. MR-Egger analysis did not suggest any directional pleiotropy for the IVs of all types of MMPs (*P* for intercept > 0.1 in both experimental and validation datasets). MR-PRESSO global test did not detect any outliers and pleiotropy, either. For heterogeneity analysis, Cochran's Q test did not detect the heterogeneity in MMP-1 (*P* > 0.10), MMP-7 (*P* > 0.05), MMP-10 (*P* > 0.10), and MMP-12 (*P* > 0.10) whereas data of MMP-3 (*P* < 0.01) were significantly heterogenous. Both of the result of MR-Egger and IVW method were consistent in heterogeneity analysis.

**Table 1:** MR results of the causal effect of MMP-1 on ER-negative BC.

Exposure	Outcome	No. of SNPs	Method	OR (95% CI)	*P *value	Heterogeneity		Pleiotropy
						Cochrane's Q	*P*	*P*
MMP1	ER- Breast cancer (Experimental set)	15	MR Egger	0.94(0.85–1.05)	0.29	14.24	0.36	0.64
			Weighted median	0.91(0.86–0.97)	< 0.01			
			Inverse variance weighted	0.92(0.88–0.97)	< 0.01	14.50	0.41	
			Simple mode	0.89(0.80–1.00)	0.07			
			Weighted mode	0.91(0.86–0.97)	0.01			
MMP1	ER- Breast cancer (Validation set1)	15	MR egger	0.95(0.83–1.09)	0.50	12.44	0.49	0.71
			Weighted median	0.90(0.83–0.98)	0.01			
			Inverse variance weighted	0.93(0.87–0.99)	0.03	12.59	0.56	
			Simple mode	0.91(0.78–1.06)	0.25			
			Weighted mode	0.90(0.82–0.98)	0.03			
MMP1	ER- Breast cancer (Validation set2)	15	MR Egger	0.97(0.82–1.15)	0.72	13.35	0.42	0.54
			Weighted median	0.94(0.85–1.04)	0.21			
			Inverse variance weighted	0.92(0.86–1.00)	0.05	13.77	0.47	
			Simple mode	0.96(0.79–1.16)	0.69			
			Weighted mode	0.95(0.85–1.05)	0.33			
MMP1	ER- Breast cancer (Validation set3)	15	MR Egger	0.86(0.67–1.11)	0.27	10.32	0.67	0.72
			Weighted median	0.91(0.79–1.05)	0.19			
			Inverse variance weighted	0.90(0.80–1.11)	0.08	10.46	0.73	
			Simple mode	1.03(0.80–1.35)	0.78			
			Weighted mode	0.92(0.80–1.05)	0.23			
MMP1	ER- Breast cancer (Validation set4)	15	MR Egger	0.94(0.80–1.10)	0.43	18.25	0.20	0.79
			Weighted median	0.92(0.84–1.02)	0.12			
			Inverse variance weighted	0.92(0.85–1.00)	0.05	18.34	0.24	
			Simple mode	0.83(0.67–1.03)	0.11			
			Weighted mode	0.92(0.83–1.02)	0.15			

*BC* Breast cancer; *CI* Confidence interval; *ER* Estrogen receptor; *MMP* Matrix metalloproteinases; *MR* Mendelian randomization; *OR* Odds ratio; *SNP* Single nucleotide polymorphisms.

**Table 2:** MR results of the causal effect of MMP-3 on ER-negative BC.

Exposure	Outcome	No. of SNPs	Method	OR (95% CI)	*P* value	Heterogeneity		Pleiotropy
						Cochrane's Q	*P*	*P*
MMP3	ER- Breast cancer (Experimental set)	12	MR Egger	1.08(0.91–1.29)	0.39	35.30	< 0.01	0.37
			Weighted median	1.03(0.96–1.11)	0.38			
			Inverse variance weighted	1.01(0.93–1.09)	0.88	38.39	< 0.01	
			Simple mode	0.94(0.81–1.09)	0.42			
			Weighted mode	1.04(0.98–1.10)	0.22			
MMP3	ER- Breast cancer (Validation set1)	12	MR Egger	1.12(0.95–1.31)	0.20	15.01	0.13	0.13
			Weighted median	1.02(0.92–1.13)	0.74			
			Inverse variance weighted	0.99(0.91–1.08)	0.88	19.16	0.06	
			Simple mode	0.93(0.76–1.14)	0.49			
			Weighted mode	1.06(0.97–1.16)	0.24			
MMP3	ER- Breast cancer (Validation set2)	12	MR Egger	1.00(0.73–1.37)	0.98	39.28	< 0.01	0.96
			Weighted median	1.02(0.93–1.13)	0.63			
			Inverse variance weighted	0.99(0.86–1.14)	0.88	39.29	< 0.01	
			Simple mode	1.01(0.84–1.21)	0.92			
			Weighted mode	1.02(0.94–1.11)	0.62			
MMP3	ER- Breast cancer (Validation set3)	12	MR Egger	1.15(0.91–1.46)	0.26	8.84	0.55	0.54
			Weighted median	1.08(0.95–1.23)	0.27			
			Inverse variance weighted	1.08(0.97–1.20)	0.17	9.25	0.60	
			Simple mode	0.97(0.75–1.26)	0.83			
			Weighted mode	1.07(0.93–1.24)	0.36			
MMP3	ER- Breast cancer (Validation set4)	12	MR Egger	1.00(0.78–1.29)	0.97	24.54	< 0.01	0.97
			Weighted median	1.01(0.92–1.12)	0.81			
			Inverse variance weighted	1.00(0.89–1.12)	1.00	24.55	0.01	
			Simple mode	1.00(0.83–1.21)	0.99			
			Weighted mode	1.02(0.93–1.12)	0.61			

*BC* breast cancer; CI, confidence interval; *ER* estrogen receptor; *MMP* Matrix metalloproteinases; *MR* Mendelian randomization; *OR* Odds ratio; *SNP* Single nucleotide polymorphisms.

**Table 3:** MR results of the causal effect of MMP-7 on ER-negative BC.

Exposure	Outcome	No. of SNPs	Method	OR (95% CI)	*P*-value	Heterogeneity		Pleiotropy
						Cochrane's Q	*P*	*P*
MMP7	ER- Breast cancer (Experimental set)	9	MR Egger	1.02(0.90–1.17)	0.73	12.23	0.09	0.61
			Weighted median	1.03(0.96–1.12)	0.38			
			Inverse variance weighted	1.05(0.97–1.14)	0.24	12.73	0.12	
			Simple mode	1.02(0.85–1.22)	0.83			
			Weighted mode	1.03(0.96–1.12)	0.43			
MMP7	ER- Breast cancer (Validation set1)	9	MR Egger	0.99(0.86–1.14)	0.89	4.99	0.66	0.79
			Weighted median	1.00(0.90–1.11)	0.97			
			Inverse variance weighted	0.98(0.89–1.07)	0.60	5.07	0.75	
			Simple mode	0.94(0.78–1.14)	0.57			
			Weighted mode	0.99(0.88–1.12)	0.92			
MMP7	ER- Breast cancer (Validation set2)	9	MR Egger	1.05(0.85–1.29)	0.67	11.34	0.12	0.60
			Weighted median	1.06(0.92–1.22)	0.41			
			Inverse variance weighted	1.10(0.96–1.26)	0.19	11.84	0.16	
			Simple mode	1.20(0.86–1.68)	0.32			
			Weighted mode	1.04(0.90–1.21)	0.57			
MMP7	ER- Breast cancer (Validation set3)	9	MR Egger	1.08(0.79–1.47)	0.66	10.88	0.14	0.35
			Weighted median	1.13(0.93–1.39)	0.23			
			Inverse variance weighted	1.22(0.99–1.50)	0.06	12.47	0.13	
			Simple mode	1.22(0.84–1.78)	0.34			
			Weighted mode	1.16(0.95–1.41)	0.19			
MMP7	ER- Breast cancer (Validation set4)	9	MR Egger	1.05(0.84–1.32)	0.66	12.65	0.08	0.76
			Weighted median	1.04(0.90–1.19)	0.61			
			Inverse variance weighted	1.08(0.94–1.25)	0.28	12.82	0.12	
			Simple mode	1.04(0.77–1.40)	0.82			
			Weighted mode	1.03(0.89–1.18)	0.73			

*BC* breast cancer; CI, confidence interval; *ER* estrogen receptor; *MMP* Matrix metalloproteinases; *MR* Mendelian randomization; *OR* Odds ratio; *SNP* Single nucleotide polymorphisms.

**Table 4:** MR results of the causal effect of MMP-10 on ER-negative BC.

Exposure	Outcome	No. of SNPs	Method	OR (95% CI)	*P* value	Heterogeneity		Pleiotropy
						Cochrane's Q	*P*	*P*
MMP10	ER- Breast cancer (Experimental set)	9	MR Egger	1.01(0.92–1.10)	0.89	10.65	0.16	0.97
			Weighted median	1.02(0.96–1.09)	0.58			
			Inverse variance weighted	1.00(0.95–1.06)	0.86	10.65	0.22	
			Simple mode	1.03(0.92–1.15)	0.64			
			Weighted mode	1.02(0.95–1.09)	0.60			
MMP10	ER- Breast cancer (Validation set1)	9	MR Egger	1.00(0.90–1.11)	0.99	7.39	0.39	0.86
			Weighted median	1.00(0.91–1.09)	0.93			
			Inverse variance weighted	0.99(0.93–1.06)	0.86	7.43	0.49	
			Simple mode	0.98(0.85–1.12)	0.74			
			Weighted mode	1.01(0.93–1.10)	0.79			
MMP10	ER- Breast cancer (Validation set2)	9	MR Egger	1.01(0.83–1.23)	0.93	17.54	0.01	0.90
			Weighted median	1.00(0.90–1.12)	0.96			
			Inverse variance weighted	1.00(0.89–1.13)	1.00	17.59	0.03	
			Simple mode	1.00(0.81–1.24)	0.97			
			Weighted mode	1.00(0.89–1.12)	0.94			
MMP10	ER- Breast cancer (Validation set3)	9	MR Egger	1.02(0.84–1.24)	0.86	8.50	0.29	0.68
			Weighted median	1.02(0.87–1.20)	0.78			
			Inverse variance weighted	1.05(0.93–1.19)	0.43	8.73	0.37	
			Simple mode	1.19(0.95–1.49)	0.17			
			Weighted mode	1.06(0.90–1.24)	0.52			
MMP10	ER- Breast cancer (Validation set4)	9	MR Egger	1.01(0.83–1.22)	0.93	16.88	0.02	0.92
			Weighted median	1.01(0.90–1.13)	0.86			
			Inverse variance weighted	1.00(0.89–1.13)	0.98	16.90	0.03	
			Simple mode	1.04(0.83–1.29)	0.74			
			Weighted mode	1.01(0.90–1.14)	0.84			

*BC* breast cancer; CI, confidence interval; *ER* estrogen receptor; *MMP* Matrix metalloproteinases; *MR* Mendelian randomization; *OR* Odds ratio; *SNP* Single nucleotide polymorphisms.

**Table 5:** MR results of the causal effect of MMP-12 on ER-negative BC.

Exposure	Outcome	No. of SNPs	Method	OR (95% CI)	*P* value	Heterogeneity		Pleiotropy
						Cochrane's Q	*P*	*P*
MMP12	ER- Breast cancer (Experimental set)	14	MR Egger	0.98(0.88–1.08)	0.64	17.70	0.13	0.39
			Weighted median	1.01(0.96–1.07)	0.70			
			Inverse variance weighted	1.02(0.96–1.07)	0.56	18.89	0.13	
			Simple mode	0.98(0.89–1.09)	0.77			
			Weighted mode	1.01(0.96–1.06)	0.78			
MMP12	ER- Breast cancer (Validation set1)	14	MR Egger	1.05(0.93–1.18)	0.46	11.79	0.46	0.74
			Weighted median	1.05(0.98–1.14)	0.18			
			Inverse variance weighted	1.07(1.00–1.13)	0.04	11.91	0.53	
			Simple mode	1.05(0.92–1.19)	0.49			
			Weighted mode	1.06(0.98–1.14)	0.20			
MMP12	ER- Breast cancer (Validation set2)	14	MR Egger	0.91(0.79–1.06)	0.25	10.41	0.58	0.31
			Weighted median	0.98(0.90–1.08)	0.73			
			Inverse variance weighted	0.98(0.91–1.05)	0.59	11.56	0.56	
			Simple mode	0.88(0.73–1.06)	0.21			
			Weighted mode	0.98(0.90–1.07)	0.69			
MMP12	ER- Breast cancer (Validation set3)	14	MR Egger	0.91(0.73–1.14)	0.43	13.92	0.31	0.68
			Weighted median	0.93(0.82–1.05)	0.25			
			Inverse variance weighted	0.95(0.85–1.06)	0.35	14.12	0.37	
			Simple mode	0.98(0.76–1.25)	0.86			
			Weighted mode	0.95(0.84–1.08)	0.45			
MMP12	ER- Breast cancer (Validation set4)	14	MR Egger	0.90(0.78–1.04)	0.18	11.92	0.45	0.15
			Weighted median	1.00(0.91–1.08)	0.86			
			Inverse variance weighted	1.00(0.92–1.07)	0.86	14.26	0.36	
			Simple mode	1.00(0.81–1.18)	0.83			
			Weighted mode	1.00(0.90–1.08)	0.77			

*BC* breast cancer; CI, confidence interval; *ER* estrogen receptor; *MMP* Matrix metalloproteinases; *MR* Mendelian randomization; *OR* Odds ratio; *SNP* Single nucleotide polymorphisms.

**Figure 1:**
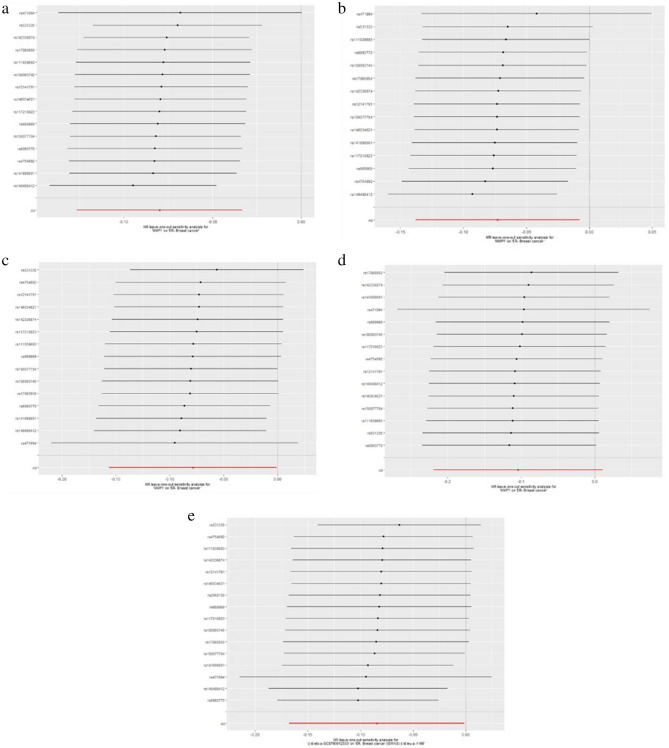
Leave-one-out plots for analysis of causal effect of MMP-1 on ER-negative BC. (**a**) Associations between MMP-1 and ER-negative BC (experimental set: ieu-a-1128); (**b**) Associations between MMP-1 and ER-negative BC (Validation set 1: ieu-a-1135); (**c**) Associations between MMP-1 and ER-negative BC (Validation set 2: ieu-a-1136); (**d**) Associations between MMP-1 and ER-negative BC (Validation set 3: ieu-a-1137); (**e**) Associations between MMP-1 and ER-negative BC (Validation set 4: ieu-a-1166). MMP, matrix metalloproteinases; ER-negative BC, estrogen receptor-negative breast cancer.

**Figure 2:**
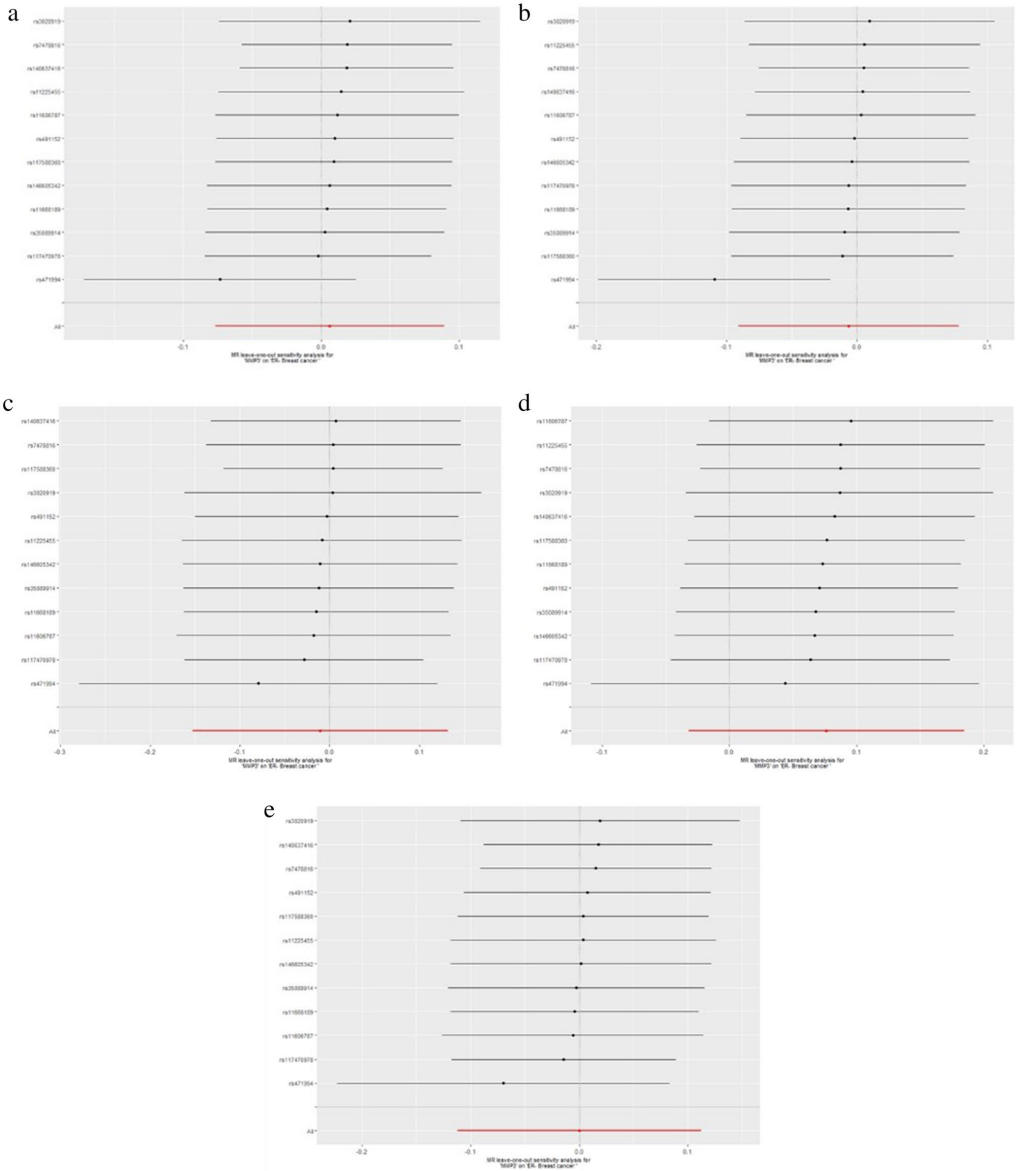
Leave-one-out plots for analysis of causal effect of MMP-3 on ER-negative BC.(**a**) Associations between MMP-3 and ER-negative BC (experimental set: ieu-a-1128); (**b**) Associations between MMP-3 and ER-negative BC (Validation set 1: ieu-a-1135); (**c**) Associations between MMP-3 and ER-negative BC (Validation set 3: ieu-a-1136); (**d**) Associations between MMP-3 and ER-negative BC (Validation set 3: ieu-a-1137); (**e**) Associations between MMP-3 and ER-negative BC (Validation set 4: ieu-a-1166), MMP, matrix metalloproteinases; ER-negative BC, estrogen receptor-negative breast cancer.

**Figure 3:**
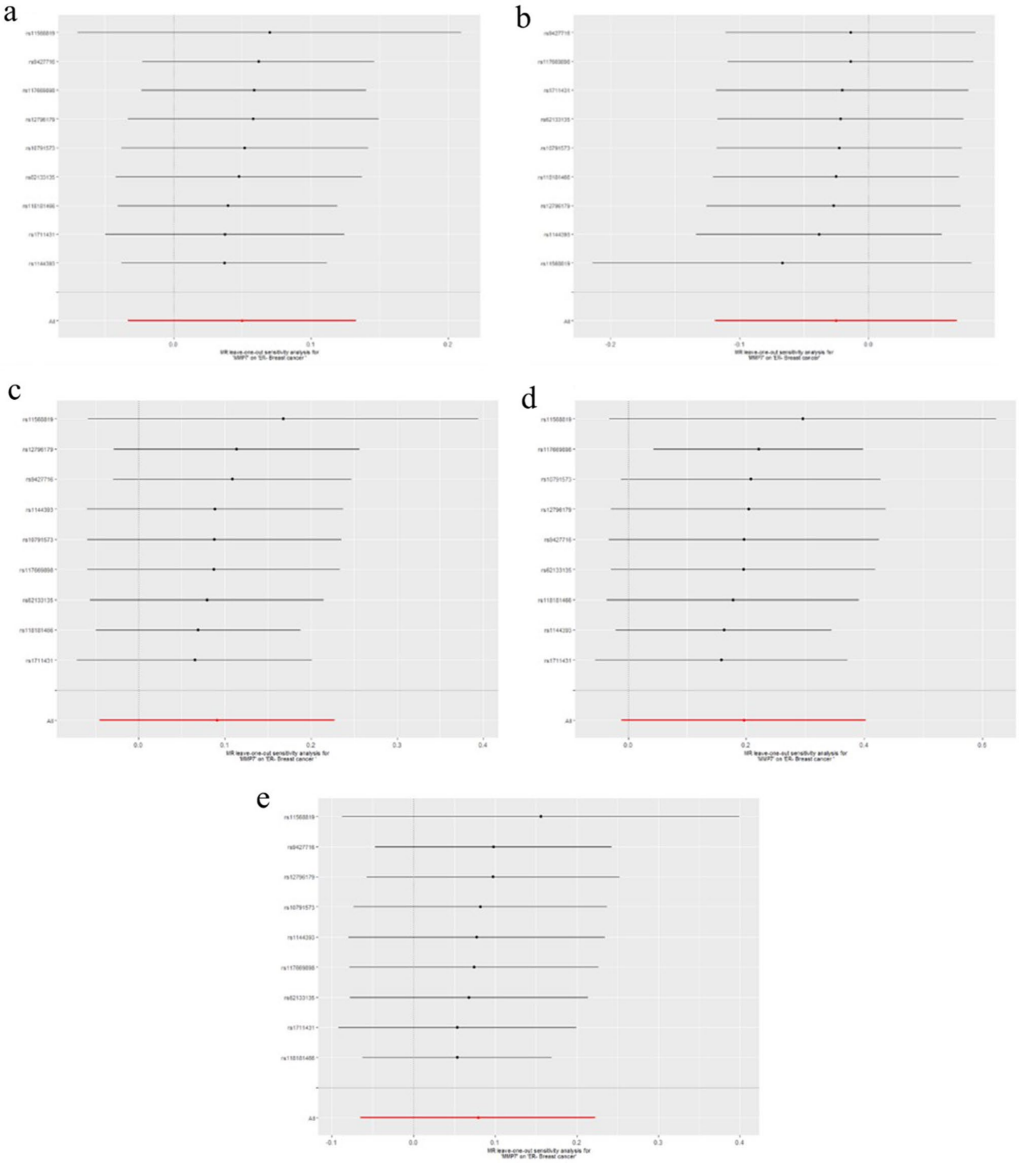
Leave-one-out plots for analysis of causal effect of MMP-7 on ER-negative BC. (**a**) Associations between MMP-7 and ER-negative BC (experimental set: ieu-a-1128); (**b**) Associations between MMP-7 and ER-negative BC (Validation set 1: ieu-a-1135); (**c**) Associations between MMP-7 and ER-negative BC (Validation set 3: ieu-a-1136); (**d**) Associations between MMP-7 and ER-negative BC (Validation set 3: ieu-a-1137); (**e**) Associations between MMP-7 and ER-negative BC (Validation set 4: ieu-a-1166), MMP, matrix metalloproteinases; ER-negative BC, estrogen receptor-negative breast cancer.

**Figure 4:**
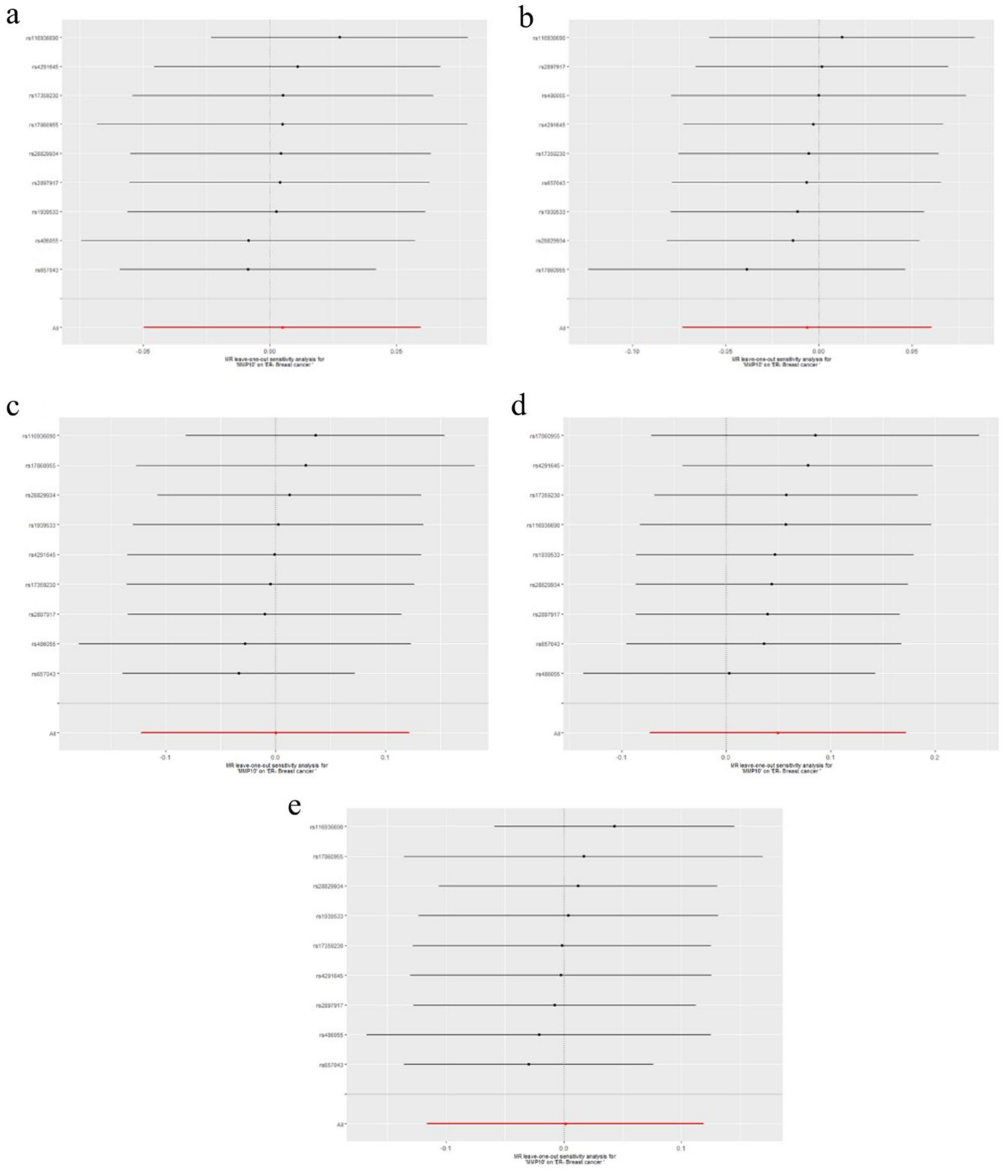
Leave-one-out plots for analysis of causal effect of MMP-10 on ER-negative BC. (**a**) Associations between MMP-10 and ER-negative BC (experimental set: ieu-a-1128); (**b**) Associations between MMP-10 and ER-negative BC (Validation set 1: ieu-a-1135); (**c**) Associations between MMP-10 and ER-negative BC (Validation set 3: ieu-a-1136); (**d**) Associations between MMP-10 and ER-negative BC (Validation set 3: ieu-a-1137); (**e**) Associations between MMP-10 and ER-negative BC (Validation set 4: ieu-a-1166), MMP, matrix metalloproteinases; ER-negative BC, estrogen receptor-negative breast cancer.

**Figure 5:**
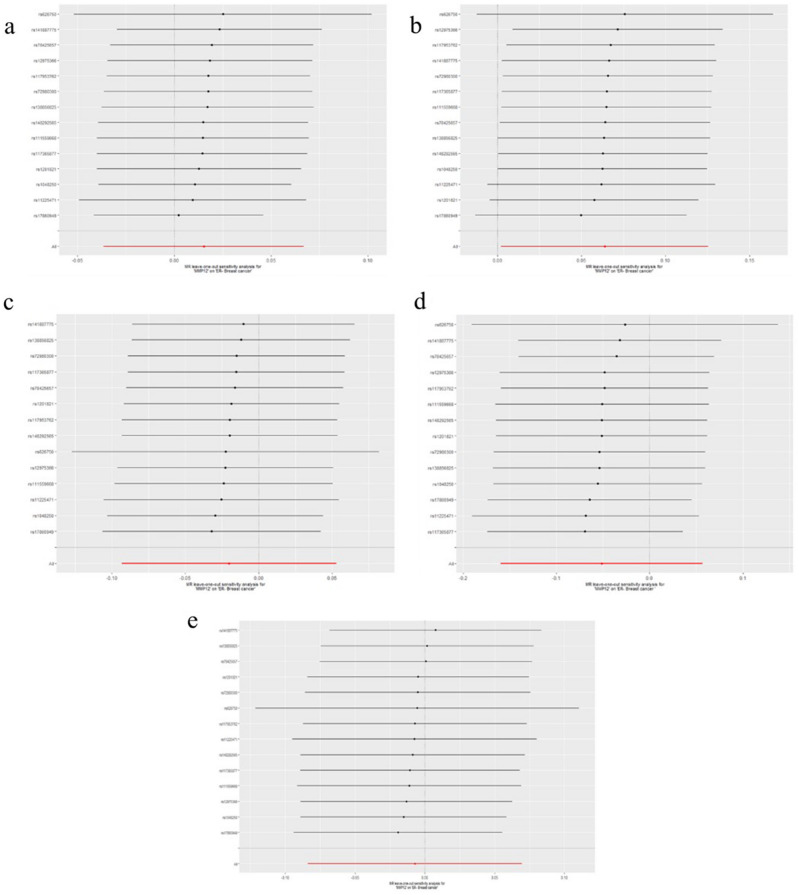
Leave-one-out plots for analysis of causal effect of MMP-12 on ER-negative BC. (**a**) Associations between MMP-12 and ER-negative BC (experimental set: ieu-a-1128); (**b**) Associations between MMP-12 and ER-negative BC (Validation set 1: ieu-a-1135); (**c**) Associations between MMP-12 and ER-negative BC (Validation set 3: ieu-a-1136); (**d**) Associations between MMP-12 and ER-negative BC (Validation set 3: ieu-a-1137); (**e**) Associations between MMP-12 and ER-negative BC (Validation set 4: ieu-a-1166). MMP, matrix metalloproteinases; ER-negative BC, estrogen receptor-negative breast cancer.

The results of leave-one-out sensitivity analysis showed that no SNPs with potential effect on the pooled result in analysis of experimental datasets Figs. 1, 2, 3, 4, 5. Scatter plots and funnel plots for analysis of MMP and BC in both experimental and validation sets are presented in Supplementary Figure (Figs. S1, S2, S3, S4, S5 for scatter plots, S6–S10 for forest plots, and S11–S15 for funnel plots).

### Causal effect of on ER-negative BC on serum MMP levels

**Figure 6:**
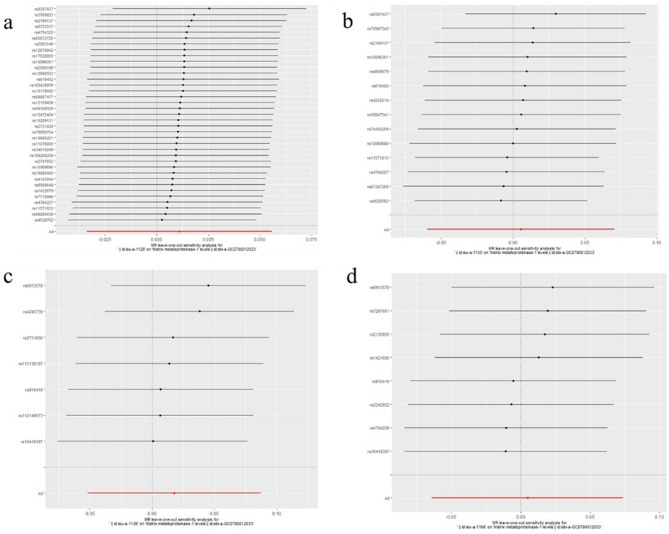
Leave-one-out plots for analysis of causal effect of ER-negative BC on MMP-1. (**a**) Associations between ER-negative BC (ieu-a-1128) and MMP-1; (**b**) Associations between ER-negative BC (ieu-a-1135) and MMP-1; (**c**) Associations between ER-negative BC (ieu-a-1136) and MMP-1; (**d**) Associations between ER-negative BC (ieu-a-1166) and MMP-1, MMP, matrix metalloproteinases; ER-negative BC, estrogen receptor-negative breast cancer.

**Figure 7:**
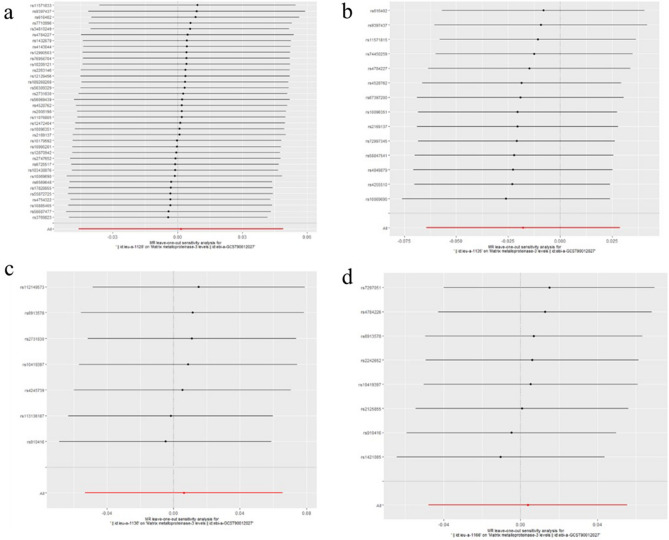
Leave-one-out plots for analysis of causal effect of ER-negative BC on MMP-3. (**a**) Associations between ER-negative BC (ieu-a-1128) and MMP-3; (**b**) Associations between ER-negative BC (ieu-a-1135) and MMP-3; (**c**) Associations between ER-negative BC (ieu-a-1136) and MMP-3; (**d**) Associations between ER-negative BC (ieu-a-1166) and MMP-3. MMP, matrix metalloproteinases; ER-negative BC, estrogen receptor-negative breast cancer.

**Figure 8:**
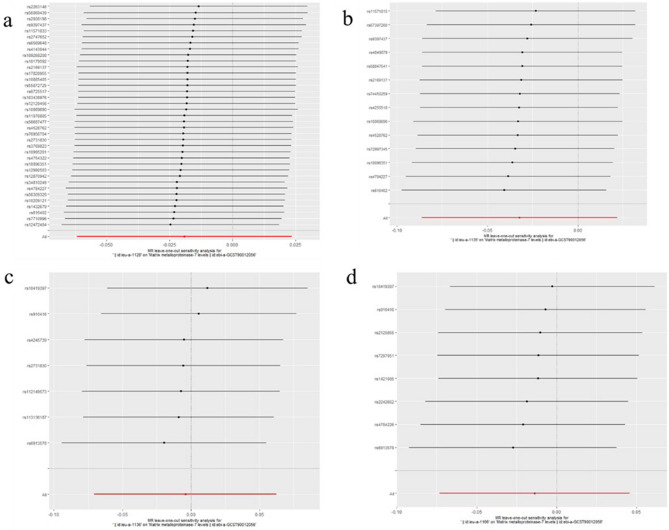
Leave-one-out plots for analysis of causal effect of ER-negative BC on MMP-7. (**a**) Associations between ER-negative BC (ieu-a-1128) and MMP-7; (**b**) Associations between ER-negative BC (ieu-a-1135) and MMP-7; (**c**) Associations between ER-negative BC (ieu-a-1136) and MMP-7; (**d**) Associations between ER-negative BC (ieu-a-1166) and MMP-7.MMP, matrix metalloproteinases; ER-negative BC, estrogen receptor-negative breast cancer.

**Figure 9:**
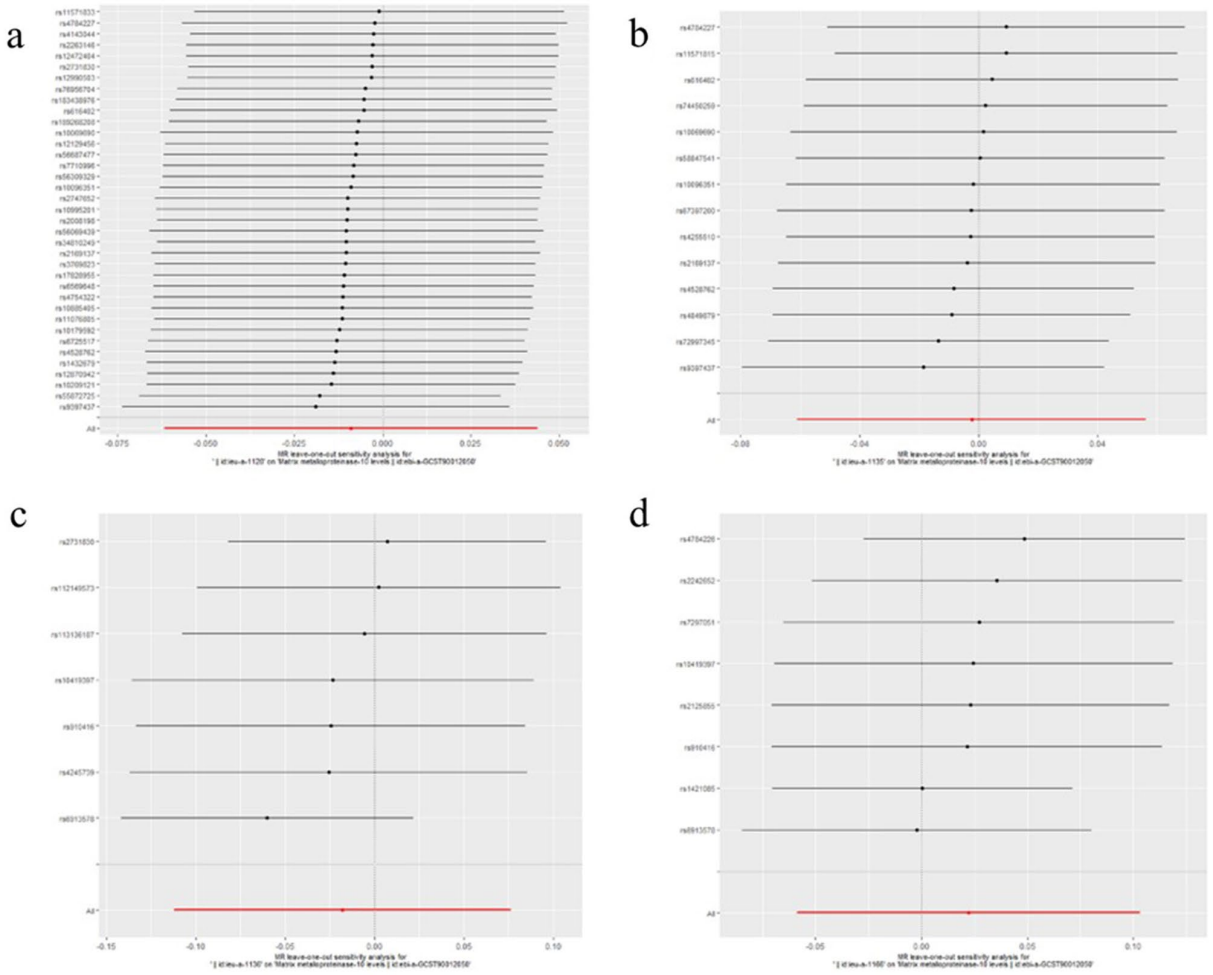
Leave-one-out plots for analysis of causal effect of ER-negative BC on MMP-10. (**a**) Associations between ER-negative BC (ieu-a-1128) and MMP-10; (**b**) Associations between ER-negative BC (ieu-a-1135) and MMP-10; (**c**) Associations between ER-negative BC (ieu-a-1136) and MMP-10; (**d**) Associations between ER-negative BC (ieu-a-1166) and MMP-10.MMP, matrix metalloproteinases; ER-negative BC, estrogen receptor-negative breast cancer.

**Figure 10:**
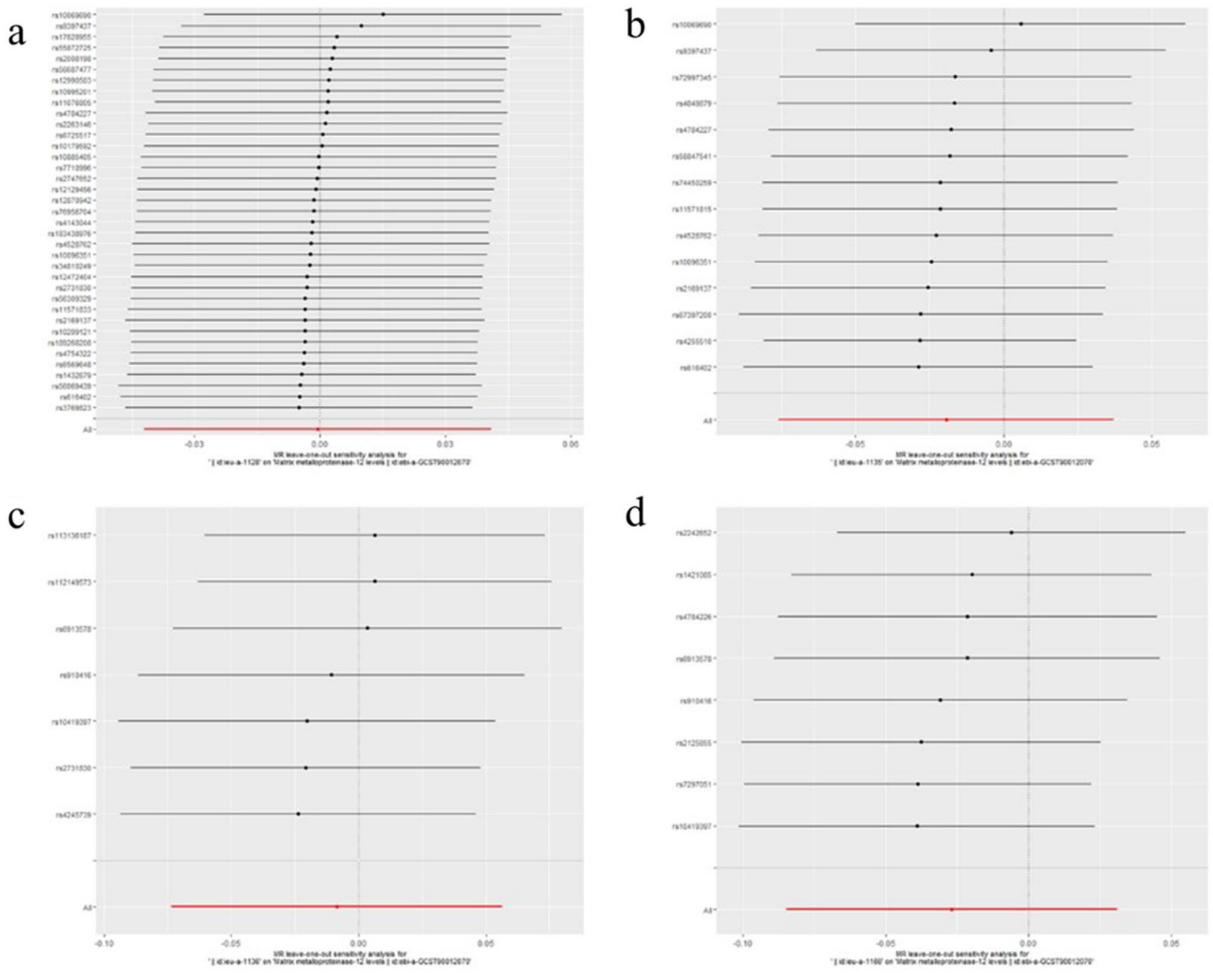
Leave-one-out plots for analysis of causal effect of ER-negative BC on MMP-12. (**a**) Associations between ER-negative BC (ieu-a-1128) and MMP-12; (**b**) Associations between ER-negative BC (ieu-a-1135) and MMP-12; (**c**) Associations between ER-negative BC (ieu-a-1136) and MMP-12; (**d**) Associations between ER-negative BC (ieu-a-1166) and MMP-12, MMP, matrix metalloproteinases; ER-negative BC, estrogen receptor-negative breast cancer.

To evaluate reverse causation effects, we planned to use the above five GWAS summary data of ER-negative BC. In the five BC GWAS data, no SNP potentially associated with confounders was removed. For the five GWAS summary datasets of ER-negative BC, the first one (GWAS ID: ieu-a-1128) has 40 exposure-associated SNPs, the second one (GWAS ID: ieu-a-1135) has 14 exposure-associated SNPs, The third one (GWAS ID: ieu-a-1136) has seven SNPs, the fourth one (GWAS ID: ieu-a-1137) has only 2 significantly related SNPs, and the last one (GWAS ID: ieu- a-1166) has eight SNPs. Because the number of selected SNP in the fourth dataset (GWAS ID: ieu-a-1137) was less than five, we used the other four datasets to investigate potential causal effect of ER-negative BC on serum level of the five MMPs. Using IVW method, neither of the results derived from these datasets indicated causality from ER-negative BC to the serum level of the five kinds of MMPs (For MMP-1, GWAS ID: ieu-a-1128: *P*= 0.63; GWAS ID: ieu-a-1135: *P*=0.87; GWAS ID: ieu-a-1136: *P*= 0.61; GWAS ID: ieu-a-1166: *P*= 0.89; For MMP-3, GWAS ID: ieu-a-1128: *P*= 0.95; GWAS ID: ieu-a-1135: *P*=0.45; GWAS ID: ieu-a-1136: *P*= 0.84; GWAS ID: ieu-a-1166: *P*= 0.88; For MMP-7, GWAS ID: ieu-a-1128: *P*= 0.38; GWAS ID: ieu-a-1135: *P*=0.24; GWAS ID: ieu-a-1136: *P*= 0.90; GWAS ID: ieu-a-1166: *P*= 0.65; For MMP-10, GWAS ID: ieu-a-1128: *P*= 0.74; GWAS ID: ieu-a-1135: *P*=0.94; GWAS ID: ieu-a-1136: *P*= 0.71; GWAS ID: ieu-a-1166: *P*= 0.59; For MMP-12, GWAS ID: ieu-a-1128: *P*= 0.98; GWAS ID: ieu-a-1135: *P*=0.50; GWAS ID: ieu-a-1136: *P*= 0.80; GWAS ID: ieu-a-1166: *P*= 0.36)**.** These results were also supported by the other four methods (MR-Egger, Weighted median, simple mode, and weighted mode). No pleiotropy (*P*>0.4) or outlier was detected by sensitivity analysis throughout the analysis (Tables 6, 7, 8, 9, 10). No remarkable heterogeneity was found either by MR-Egger or IVW methods for analysis of causal effect of BC on MMP-1/-7/-10/-12 (*P*>0.05) but MMP-3 (In GWAS data ieu-a-1128: MR Egger: *P*=0.03; IVW method: *P*=0.01) (Table 7**)**. For analysis between ER-negative BC (GWAS data ieu-a-1128) and the five types of MMPs, three SNPs were removed for being palindromic with intermediate allele frequencies: rs2735846, rs62116991, and rs191981806. As a result, the results were derived from the remaining 37 SNPs. Leave-one-out plots indicated that no SNP in all four GWAS summary datasets of ER-negative BC had great impact on the MR analysis (Figs. 6, 7, 8, 9, 10). F-statistic for SNPs of all the four datasets of ER-negative BC were greater than the threshold of 10, suggesting strong IVs, which reducing bias of IVs estimates (Supplementary Tables S6, S7, S8, S9, the F statistics for analysis with the other four types of MMPs were the same as the analysis with MMP-1).

**Table 6:** MR results of the causal effect of ER-negative BC on MMP-1.

Exposure	Outcome	No. of SNPs	Method	β	*P* value	Heterogeneity		Pleiotropy
						Cochrane's Q	*P*	*P*
ER-negative BC (ieu-a-1128)	MMP1	37	MR Egger	0.06	0.35	35.13	0.46	0.39
			Weighted median	0.03	0.37			
			Inverse variance weighted	0.01	0.63	35.81	0.48	
			Simple mode	− 0.06	0.40			
			Weighted mode	0.04	0.54			
ER-negative BC (ieu-a-1135)	MMP1	14	MR Egger	− 0.04	0.71	16.86	0.15	0.74
			Weighted median	− 0.001	0.98			
			Inverse variance weighted	0.005	0.87	17.17	0.19	
			Simple mode	− 0.03	0.64			
			Weighted mode	0.03	0.66			
ER-negative BC (ieu-a-1136)	MMP1	7	MR Egger	0.009	0.96	5.48	0.36	0.31
			Weighted median	0.05	0.28			
			Inverse variance weighted	0.02	0.61	5.48	0.48	
			Simple mode	0.08	0.33			
			Weighted mode	0.09	0.35			
ER-negative BC (ieu-a-1166)	MMP1	8	MR Egger	0.17	0.54	8.01	0.24	0.68
			Weighted median	0.02	0.68			
			Inverse variance weighted	0.005	0.89	8.56	0.29	
			Simple mode	0.10	0.32			
			Weighted mode	0.10	0.31			

*BC* Breast cancer; *ER* Estrogen receptor; *MMP* Matrix metalloproteinases; *MR* Mendelian randomization; *SNP* Single nucleotide polymorphisms.

**Table 7:** MR results of the causal effect of ER-negative BC on MMP-3.

Exposure	Outcome	No. of SNPs	Method	β	*P* value	Heterogeneity		Pleiotropy
						Cochrane's Q	*P*	*P*
ER-negative BC (ieu-a-1128)	MMP3	37	MR Egger	− 0.12	0.07	52.17	0.03	0.04
			Weighted median	− 0.005	0.86			
			Inverse variance weighted	0.002	0.95	58.61	0.01	
			Simple mode	− 0.05	0.35			
			Weighted mode	− 0.02	0.52			
ER-negative BC (ieu-a-1135)	MMP3	14	MR Egger	− 0.11	0.15	9.64	0.65	0.20
			Weighted median	− 0.008	0.81			
			Inverse variance weighted	− 0.02	0.45	11.51	0.57	
			Simple mode	0.02	0.64			
			Weighted mode	0.002	0.97			
ER-negative BC (ieu-a-1136)	MMP3	7	MR Egger	− 0.07	0.64	2.14	0.83	0.61
			Weighted median	− 0.007	0.86			
			Inverse variance weighted	0.006	0.84	2.44	0.88	
			Simple mode	− 0.02	0.78			
			Weighted mode	− 0.01	0.83			
ER-negative BC (ieu-a-1166)	MMP3	8	MR Egger	− 0.35	0.12	2.18	0.90	0.11
			Weighted median	− 0.01	0.76			
			Inverse variance weighted	0.004	0.88	5.70	0.57	
			Simple mode	− 0.01	0.83			
			Weighted mode	− 0.01	0.83			

*BC* Breast cancer; *ER* Estrogen receptor; *MMP* Matrix metalloproteinases; *MR* Mendelian randomization; *SNP* Single nucleotide polymorphisms.

**Table 8:** MR results of the causal effect of ER-negative BC on MMP-7.

Exposure	Outcome	No. of SNPs	Method	β	*P* value	Heterogeneity		Pleiotropy
						Cochrane's Q	*P*	*P*
ER-negative BC (ieu-a-1128)	MMP7	37	MR Egger	− 0.08	0.20	21.49	0.96	0.29
			Weighted median	− 0.04	0.24			
			Inverse variance weighted	− 0.02	0.38	22.63	0.96	
			Simple mode	− 0.05	0.40			
			Weighted mode	− 0.05	0.27			
ER-negative BC (ieu-a-1135)	MMP7	14	MR Egger	− 0.12	0.17	3.49	0.99	0.29
			Weighted median	− 0.04	0.33			
			Inverse variance weighted	− 0.03	0.24	4.73	0.98	
			Simple mode	− 0.04	0.43			
			Weighted mode	− 0.05	0.33			
ER-negative BC (ieu-a-1136)	MMP7	7	MR Egger	0.07	0.63	2.03	0.84	0.60
			Weighted median	0.007	0.87			
			Inverse variance weighted	− 0.004	0.90	2.34	0.89	
			Simple mode	0.02	0.74			
			Weighted mode	0.02	0.73			
ER-negative BC (ieu-a-1166)	MMP7	8	MR Egger	0.14	0.55	2.07	0.91	0.50
			Weighted median	− 0.03	0.40			
			Inverse variance weighted	− 0.01	0.65	2.58	0.92	
			Simple mode	− 0.04	0.48			
			Weighted mode	− 0.04	0.49			

*BC* Breast cancer; *ER* Estrogen receptor; *MMP* Matrix metalloproteinases; *MR* Mendelian randomization; *SNP* Single nucleotide polymorphisms.

**Table 9:** MR results of the causal effect of ER-negative BC on MMP-10.

Exposure	Outcome	No. of SNPs	Method	β	*P* value	Heterogeneity		Pleiotropy
						Cochrane's Q	*P*	*P*
ER-negative BC (ieu-a-1128)	MMP10	37	MR Egger	− 0.13	0.08	46.00	0.10	0.08
			Weighted median	0.007	0.83			
			Inverse variance weighted	− 0.009	0.74	50.38	0.06	
			Simple mode	0.05	0.45			
			Weighted mode	0.01	0.80			
ER-negative BC (ieu-a-1135)	MMP10	14	MR Egger	0.04	0.66	13.77	0.32	0.62
			Weighted median	− 0.007	0.87			
			Inverse variance weighted	− 0.002	0.94	14.07	0.37	
			Simple mode	− 0.02	0.69			
			Weighted mode	− 0.02	0.75			
ER-negative BC (ieu-a-1136)	MMP10	7	MR Egger	− 0.32	0.12	6.67	0.25	0.13
			Weighted median	0.02	0.75			
			Inverse variance weighted	− 0.02	0.71	11.11	0.08	
			Simple mode	0.02	0.86			
			Weighted mode	0.03	0.76			
ER-negative BC (ieu-a-1166)	MMP10	8	MR Egger	− 0.44	0.15	7.75	0.26	0.13
			Weighted median	0.01	0.78			
			Inverse variance weighted	0.02	0.59	11.81	0.11	
			Simple mode	0.003	0.97			
			Weighted mode	0.004	0.96			

*BC* Breast cancer; *ER* Estrogen receptor; *MMP* Matrix metalloproteinases; *MR* Mendelian randomization; *SNP* Single nucleotide polymorphisms.

**Table 10:** MR results of the causal effect of ER-negative BC on MMP-12.

Exposure	Outcome	No. of SNPs	Method	β	*P* value	Heterogeneity		Pleiotropy
						Cochrane's Q	*P*	*P*
ER-negative BC (ieu-a-1128)	MMP12	37	MR Egger	− 0.06	0.27	35.09	0.46	0.24
			Weighted median	0.02	0.50			
			Inverse variance weighted	− 0.001	0.98	36.52	0.44	
			Simple mode	0.06	0.39			
			Weighted mode	0.04	0.46			
ER-negative BC (ieu-a-1135)	MMP12	14	MR Egger	− 0.18	0.04	10.66	0.56	0.05
			Weighted median	0.02	0.61			
			Inverse variance weighted	− 0.02	0.50	15.36	0.29	
			Simple mode	0.04	0.54			
			Weighted mode	0.05	0.54			
ER-negative BC (ieu-a-1136)	MMP12	7	MR Egger	0.03	0.87	6.09	0.30	0.83
			Weighted median	0.01	0.82			
			Inverse variance weighted	− 0.01	0.80	6.15	0.41	
			Simple mode	0.04	0.55			
			Weighted mode	0.04	0.62			
ER-negative BC (ieu-a-1166)	MMP12	8	MR Egger	− 0.09	0.70	7.09	0.31	0.78
			Weighted median	− 0.02	0.62			
			Inverse variance weighted	− 0.03	0.36	7.19	0.41	
			Simple mode	0.03	0.71			
			Weighted mode	0.02	0.73			

*BC* Breast cancer; *ER* Estrogen receptor; *MMP* Matrix metalloproteinases; *MR* Mendelian randomization; *SNP* Single nucleotide polymorphisms.

## Discussion

In our study, we found that serum MMP-1 is a protective factor for ER-negative BC. In other words, a reduction of serum MMP-1 concentration had causal effect on the risk of ER-negative BC. In the opposite direction, no causal effect was found from ER-negative BC to the serum MMP-1 level. Different from MMP-1, no mutual causal relationship between the other four types of MMPs (MMP-3, MMP-7, MMP-10, and MMP-12) and ER-negative MMPs.

To the best of our knowledge, our study is the first study reporting the bidirectional causality between serum MMP level and BC. MMP could stimulate tumor cell migration, invasion, and metastasis through proteolysis of the extracellular matrix^16,44^. MMP-1 is a kind of interstitial collagenase which is capable of degrading type I, II, and III collagens. Previous studies have found that exosomal MMP-1 in circulation and MMP-1 expressed on BC cells empowered BCs (especially for TNBC) the potential of distal metastasis (brain, lung, etc.) and led to a poor disease-free survival^45,46^. One study found that MMP-1 expression was significantly higher in TNBC tissue than in ER-positive and HER-2-positive BC tissue. And MMP-1 expression was also enriched in metastatic BC tissue than in non-metastatic BC tissue^47^. Studies also reported that MMP-1 or their specific polymorphisms contributed to initiation and progression of BC but the association between MMP-1 level and overall survival was still controversial^48–51^. What’s more, certain studies even found that specific genetic variants of MMP-1 did not affect the risk of BC^52–54^. Different from the above results, one study suggested that serum MMP-1 level was significantly lower in BC patients than in healthy controls (*P* < 0.0001) and patients with a lower serum concentration of MMP-1 had a remarkably shorter 5-year survival^55^. And another study even demonstrated that stromal expression of MMP-1 was an independent prognostic factor for a longer overall survival (Hazard ratio = 0.528, *P* = 0.042)^56^. Nevertheless, few studies focused on association between circulating/serum MMP-1 and each subtype of BC. In our study, serum MMP-1 level had causal effect on ER-negative BC and a high level of MMP-1 serum level caused a lower risk of ER-negative BC, suggesting a protective role of MMP-1 in ER-negative BC. The result was derived not only from IVW method, but also from weighted median and weighted mode methods. In our study, all results were based on IVW method. Moreover, our results were considered robust as selected GWAS summary data of MMP-1 and ER-negative BC had a large sample size. Different types of sensitivity analysis also corroborated the strength and power of our results. According to result of MR analysis of causal effect of ER-negative BC on MMP-1, no positive result was found. This suggested that low serum level of MMP-1 caused ER-negative BC instead of that the latter one resulted in reduction of MMP-1 level. According to the status quo of the research of MMP-1 in breast cancer, inconsistent conclusions could be found in these studies mentioned in our discussion. Firstly, some studies only indicated that MMP-1 promoted carcinogenesis and metastasis of BC though whether all subtypes of BC could be empowered by MMP-1 was unclear. Secondly, most of MMP-related studies focused on tumoral or histological MMP expression. Instead, our study investigated relationship between specifically serum MMP molecules and ER-negative BC. Whether same result could happen in serum MMP should be further discussed. Lastly, studies have suggested that MMP-1 has several genetic variants (polymorphisms) and different variants could impact on prognosis of each subtype of BC in different ways^57^. In one study from the US in which most of the patients were from Hispanic and non-Hispanic white, investigators found that not all polymorphisms of serum MMP-1 were associated with prognosis of BC and different gene sequences could cause different clinical outcomes. MMP-1 rs17293761 TT genotype was not a risk factor for more advanced breast tumor^57^. Hence, our study not only corroborated research results in studies believing that MMP-1 was a protective factor but also put forward a new possibility of relationship between serum MMP-1 and ER-negative BC. Regarding that research of relationship between serum MMP-1 and BC was still lacking and the potential mechanism of this phenomenon was unclear, it is worth being furtherly explored to validate this result.

For the other four types of MMPs, we did not find any causality between each of them and ER-negative BC. For MMP-3 (Stromelysin-1) and MMP-10 (stromelysin-2), both of them degrade extracellular matrix (ECM) proteins including aggrecan, collagen types III and IV, and fibronectin^58^. The former one is not only expressed in cancer cells, but also in normal cells (endothelial cells, epithelial cells, macrophages, and stromal fibroblasts) while the latter one is merely detected in abnormal tissue including acute or chronic injury and cancer^59,60^. One study suggested that serum level of MMP-10 was significantly higher in BC patients than that in healthy control (*P* < 0.001). Median serum of MMP-3 was significantly higher in advanced BC (stage III and IV) than that in early-stage BC (stage I) (*P* = 0.018)^61^. Another study drew a different conclusion, suggesting that expression of MMP-10 was lower in BC tissue compared with adjacent normal tissue^62^. Also in the aforementioned study published by Dr. Martha L Slattery from Utah, USA, the clinical significance of MMP-3 were investigated^57^. Results showed that MMP-3 was associated with breast cancer risk only in part of Native Americann, with merely borderline significance (*P* = 0.06). For relationship between MMP polymorphism and tumor prognosis, two genetic variants of MMP-3 could drastically increased risk of tumor progression and distant metastasis. Nevertheless, these results were based on mixed population in which Hispanic and Native Amerivans predominated. Whether the results could be applied in other ethnicities should be further explored. For association between MMP-3 and prognosis of breast cancer, one study indicated that MMP-3 did not impact on overall survival but a higher level of cellular expression of MMP-3 had a significantly poorer metastasis-free survival^63^. Up till now, studies on relationship between MMP-10 and prognosis of breast cancer was not available. As a type of matrilysin, MMP-7 disrupts the structure of and degrade casein, collagen, elastin, fibronectin, gelatins, laminin, and proteoglycans^64^. Amongst, collagen IV, laminin, and proteoglycan are the major components of basement membrane^65^. Thus, the biological process of MMP-7 plays a crucial role in local invasion, lymph-node, and distal metastasis of cancer cells^66^. Studies have shown that serum MMP-7 was higher in BC patients compared with control group^67^. Another study found that BC patients with bone metastasis had a higher serum level of MMP-7, suggesting a potential circulating biomarker for BC progression towards bone metastasis^68^. In one study from Xi’an Jiaotong University, researchers illustrated that MMP-7 expression was higher in tissue from advanced breast cancer (larger focus, lymphatic metastasis, and distant metastasis) and patients with positive MMP-7 expression had a poorer 5-year survival (*P* = 0.046)^69^. On the contrary, another study reported that serum level of MMP-7 did not correlate with risk of breast tumor and it did not reduce after the removal of the tumor^70^. Currently, few study has reported positive result and conclusion for association or causal relatoinship between tissue/serum level of MMP-12 with BC. Above all, no consensus has been made on causal effect between these four types of MMP and BC. More intense investigtions in this field should be performed.

Despite the originality and a robust result of our study, some limitations are necessary to be stated: (1) Individuals of this study are from European Ancestry, results derived from selected SNPs could not directly extend to other ethnic groups; (2) Temporarily the GWAS summary data did not contain sufficient IVs to complete analysis for other types of MMPs so that MR analysis between these MMPs and BC could not be conducted; (3) Number of SNPs for the five MMPs were relatively small, especially for MMP-3, MMP-7, and MMP-10, a larger GWAS with more eligible IVs is needed to increase the power of MR analysis.

## Conclusions

To conclude, a low level of serum MMP-1 has a causal effect on a high risk of ER-negative BC in European population. In reverse analysis, no causal effect was found from ER-negative BC on the level of serum MMP-1. No evidence supported any causality between MMP-3, -7, -10, -12 and ER-negative BC in European ancestry. More intense research ought to be carried on to validate the serum MMPs as potential biomarkers and therapeutic targets in ER-negative BC.

## Methods

### Study design

**Figure 11:**
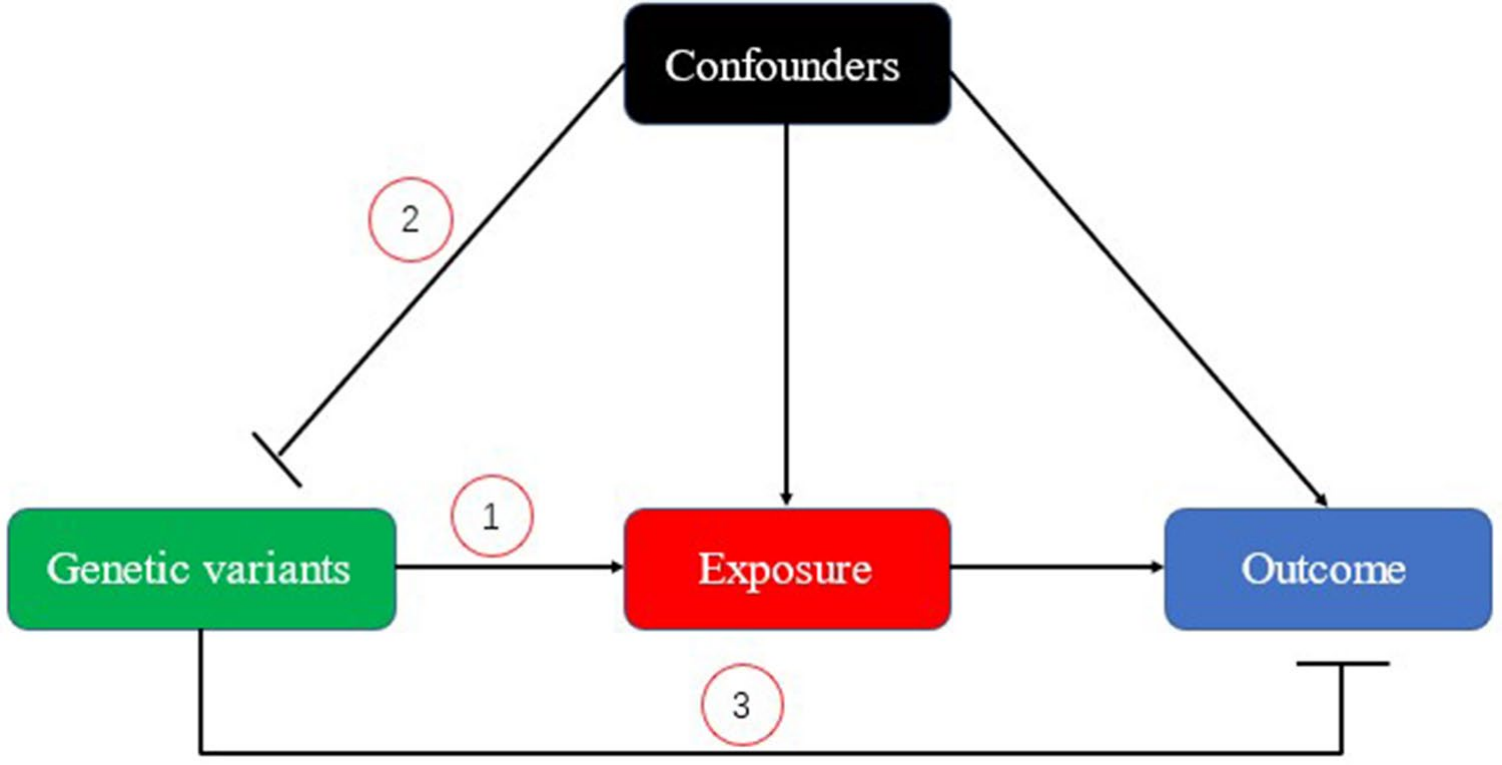
Diagram of three stringent assumptions of Mendelian randomization. Number 1, 2, and 3 stands for three main assumptions of IV selection in MR analysis. IV, instrumental variables; MR, mendelian randomization.

**Figure 12:**
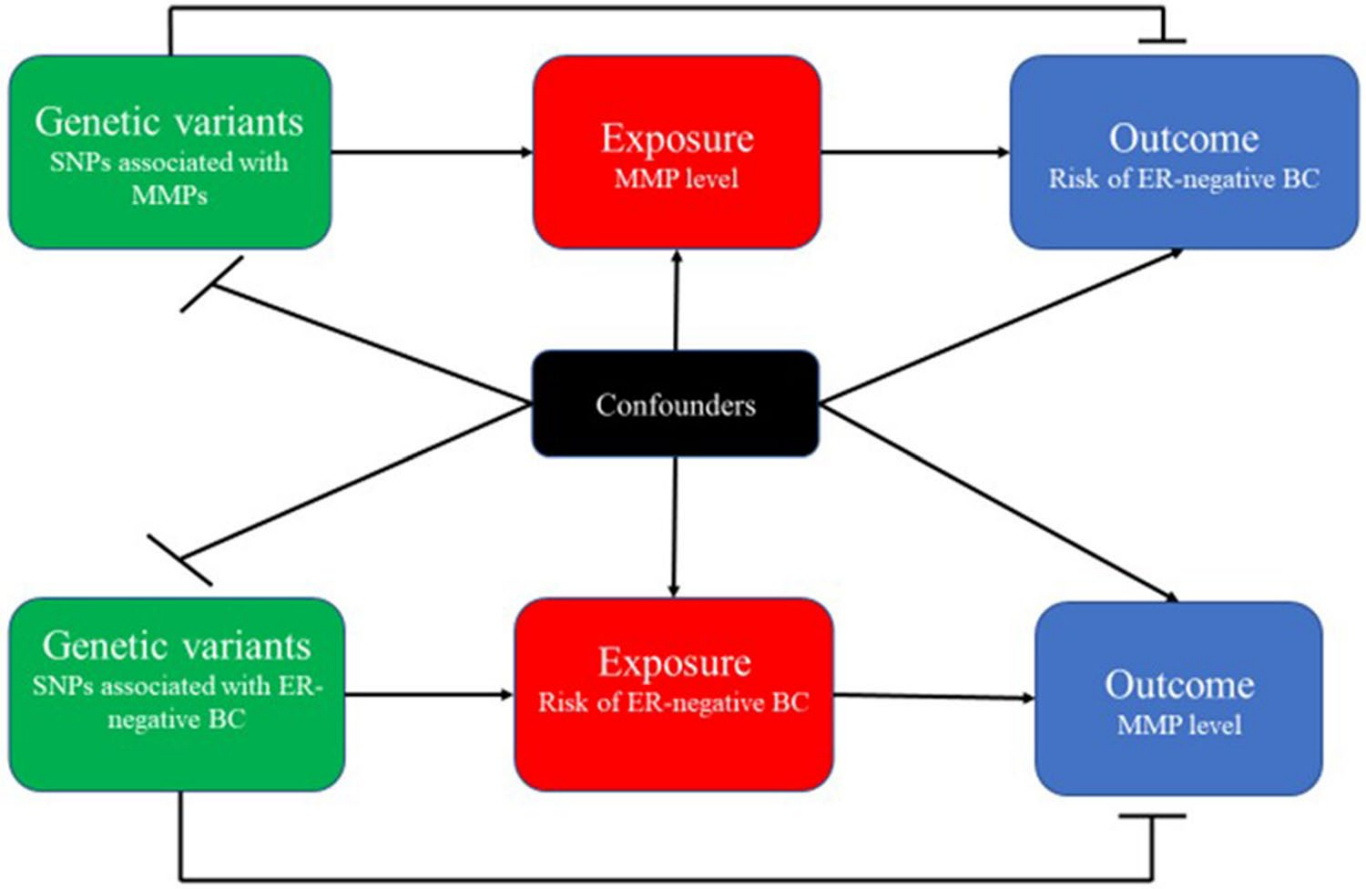
Diagram of design of bidirectional Mendelian randomization study. ER-negative BC, estrogen receptor-negative breast cancer; MMP, matrix metalloproteinases; SNP, single nucleotide polymorphisms.

Selection of IVs from genetic variants in this MR analysis strictly meet the three stringent assumptions of MR: (1) as selected IVs, the genetic variants is remarkably associated with the exposure; (2) the genetic variants is not associated with any confounding factors; (3) genetic variants could only indirectly affect the outcome via the exposure, not directly affecting or any other pathways **(**Fig. 11**)**^71^. In our study, we selected summary-level data of 5 kinds of MMPs (MMP-1,-3,-7,-10,-12, containing not less than 5 SNPs) and ER-negative BC from open database of published genome-wide association studies (GWASs) summary dataset (https://gwas.mrcieu.ac.uk/)^72,73^. We firstly collected genetic variants for each type of MMP in order to determine the causality from MMP to ER-negative BC. Then we collected genetic variants robustly associated with ER-negative BC to validate the reverse causality from BC to MMPs. This is the main goal of our study. The design of bidirectional MR study is overviewed in Fig. 12.

### Data sources and SNP selection for MMPs

Genetic variants of 5 kinds of MMPs (MMP-1, MMP-3, MMP-7, MMP-10, and MMP-12) were obtained from a meta-analysis of GWASs including 21,758 individuals from 13 cohorts of European ancestry^74^. All the five kinds of MMPs passed quality control and were normalized with rank-based inverse normal transformation and/or standardized to unit variance in order to control unrelated variables among cohorts. Genetic associations between 20.3 million genetic variants (SNPs) and log-transformed MMPs were adjusted for population structure (age, sex, smoking status, oral contraceptive usage, blood cell counts, etc.) and study-specific parameters (OLINK plate, storage time, MDS component, etc.) ^74^. To meet the first assumption of MR, we extracted the IVs of the five types of MMPs at genome-wide significance (5 × 10^−8^). 17 SNPs were significantly associated with MMP-1; 12 SNPs were significantly related with MMP-3; seven SNPs were significantly associated with MMP-7; eleven SNPs were significantly associated with MMP-10; and 15 SNPs were remarkably associated with MMP-12. Meanwhile, a linkage disequilibrium (LD) test was conducted on these SNPs to clump SNPs for independence. All SNPs were strongly and independently (R^2^ < 0.01 within 5 Mb) predicted MMP level from the published GWAS. Subsequently, we input all the SNPs significantly associated with MMPs into Phenoscanner database (V2) (http://www.phenoscanner.medschl.cam.ac.uk/) to determine if any SNPs were associated with confounders (*P* < 5 × 10^−8^)^75,76^. Resulted SNPs would be deleted to reduce the possibility of pleiotropic effect.

### Data sources and SNP selection for ER-negative BC

Summary-level data on ER-negative BC were extracted from a GWAS of 127,442 individuals of European ancestry from Breast Cancer Association Consortium (BCAC), combined with Discovery, Biology and Risk of Inherited Variants in Breast Cancer Consortium (DRIVE), iCOGS project, and data from other GWAS meta-analysis^77^. This data would be used as experimental dataset to explore potential causal effect between MMPs and ER-negative BC. Then we used the other four datasets as validation datasets to prove the conclusion draught from the experimental datasets. The four datasets were all derived from European Ancestry (OncoArray1, case: 9655, control: 45494; iCOGS, case: 7333, control: 42892; GWAS meta-analysis1, case: 4480, control: 17588; GWAS meta-analysis2: case: 3611, control: 18084)^77,78^. Similar to SNP selection for MMPs, potential SNPs correlated with confounders would be removed by using Phenoscanner database (*P*<5×10^−8^).To further evaluate robustness of selected SNPs, statistics F and R^2^ were used in both the process of SNP selection for MMP and ER-negative BC. F statistic stands for the precision and magnitude of the genetic effect on the trait. The Eq. (1) is:

1 $${\text{F}} = \, \left( {{\text{N}} - {2}} \right)*{\text{R}}^{{2}} /\left( {{1} - {\text{R}}^{{2}} } \right)$$

N stands for sample size of a certain GWAS and R^2^ is the proportion of the variance of the trait caused by genetic variants (SNPs). The Eq. (2) is:

2 $${\text{R}}^{{2}} = { 2} \times {\text{EAF}} \times \left( {{1} - {\text{EAF}}} \right) \times \beta^{{2}}$$

EAF is short for “Effect Allele Frequency” (EAF) of the SNP and β is the estimated effect of SNP on the trait. SNPs with F less than ten would be removed and SNPs with F larger than 10 were robust to prove the validity of selected SNPs

^79,80^.

### Bidirectional mendelian randomizasion analysis

Bidirectional two-sample MR was performed by using the R pacakge “TwosampleMR”. Information of SNPs, β value (created by log-transformation of odds ratios [ORs]), standard error, *P*-value, and EAF value of selected exposure instrument were necessary for this package to harmonize exposure and outcome data to investigate direction of causality between MMPs and ER-negative BC by using summary association data. In our study we did not look for proxies to replace SNPs that were not available in the outcome datasets. During data harmonization, we should ensure that all selected SNPs were derived from the same allele no matter in exposure or outcome data. For palindromic SNPs, however, they were too difficult to be recognized whether the SNPs were from the same allele because the sequence were same on both strands. As a result, palindromic SNPs were removed to eliminate the ambiguity as to whether exposure and outcome GWAS infer the same effect allele^43^. In the core process of MR analysis, we measured Wald ratio (i.e, β_outcome_/β_exposure_) for each SNP and then summarized these SNP-sepcific Wald ratio via inverse-variance-weighted (IVW) method which estimated causal effects of genetically predicted exposure on outcome^81,82^. We demonstrated the estimate effects in ORs for binary outcome (ER- negative BC) and in β for continuous outcome (MMP level). To explore the direction of causality from MMP to BC, OR was elaborated as risk for ER-negative BC (outcome) per unit increase in serum level of certain type of MMP. Other methods in MR anaysis include: MR-Egger, weighted median, simple mode, and weighted mode. A series of sensitivity analysis were performed, consisting of weighted median (WM) method, MR-Egger, and Mendelian Randomization Pleiotropy RESidual Sum and Outlier (MR-PRESSO). WM method reckons the causal effect by selecting median MR estimate for condition in which multiple genetic variants are invalid or present pleiotropy^83^. MR-Egger method is robust not only to provide a consistent estimate of causal effect, but also to evaluate horizontal pleiotropy of IVs and a non-zero intercept suggesting that the IVW estimate is biased^84,85^. MR-PRESSO is capable of detecting and correcting any potentially pleiotropic outliers (SNPs) for all reported results to avoid bias^86^. Heterogeneity was quantified by the Cochran Q statistics and I^2^ statistics, in which larger I^2^ indicates increasing heterogeneity^87^. Furthermore, “leave-one-out analysis” was also conducted by removing each SNPs to test the stability and reliability of the MR results. By virtue of multiple testing in our analysis, Bonferroni correction was used to modify the significant level for multiple tests. Thus we considered *P*-values below (0.05/25=0.002) as strong evidence of associations. Results with *P*-values between 0.002 and 0.05 were regarded as suggestive associations^43^. All statistical analysis were two-sided. All analysis was conducted using R software (4.2.0) with R package of “TwosampleMR” (version 0.5.6), “MRPRESSO” (version 1.0). Reporting follows the STROBE-MR statement.

## Supplementary Information

Supplementary Information.

## Data Availability

All data generated or analysed during this study are included in this published article and its Supplementary information files.

## Abbreviations

BC: Breast cancer
BCAC: Breast cancer association consortium
CI: Confidence interval
EAF: Effect allele frequency
ER: Estrogen receptor
GWASs: Genome-wide association studies
HER-2: Human epidermal growth factor receptor 2
IVW: Inverse-variance-weighted
IVs: Instrumental variables
LD: Linkage disequilibrium
MMP: Matrix metalloproteinases
MR: Mendelian randomization
MR-PRESSO: Mendelian randomization pleiotropy RESidual sum and outlier
OR: Odds ratio
SNP: Single nucleotide polymorphisms
TNBC: Triple-negative breast cancer

## References

[1] Sung H (2021). Global cancer statistics 2020: GLOBOCAN estimates of incidence and mortality worldwide for 36 cancers in 185 countries. CA Cancer J. Clin..

[2] Voduc KD (2010). Breast cancer subtypes and the risk of local and regional relapse. J. Clin. Oncol..

[3] Belete AM, Aynalem YA, Gemeda BN, Demelew TM, Shiferaw WS (2022). The effect of estrogen receptor status on survival in breast cancer patients in ethiopia retrospective cohort study. Breast Cancer.

[4] Davies C (2011). Relevance of breast cancer hormone receptors and other factors to the efficacy of adjuvant tamoxifen: Patient-level meta-analysis of randomised trials. Lancet.

[5] Colditz GA, Rosner BA, Chen WY, Holmes MD, Hankinson SE (2004). Risk factors for breast cancer according to estrogen and progesterone receptor status. J. Natl. Cancer Inst..

[6] Goddard KA (2012). HER2 evaluation and its impact on breast cancer treatment decisions. Public Health Genom..

[7] Howlader N (2014). US incidence of breast cancer subtypes defined by joint hormone receptor and HER2 status. J. Natl. Cancer Inst..

[8] Jerusalem G, Lancellotti P, Kim SB (2019). HER2+ breast cancer treatment and cardiotoxicity: Monitoring and management. Breast Cancer Res. Treat..

[9] Asif HM, Sultana S, Ahmed S, Akhtar N, Tariq M (2016). HER-2 positive breast cancer - a mini-review. Asian Pac. J. Cancer Prev..

[10] Parakh S, Gan HK, Scott AM (2020). Sensitization of cancers resistant to HER2 antibodies. Crit. Rev. Oncog..

[11] Bredin P, Walshe JM, Denduluri N (2020). Systemic therapy for metastatic HER2-positive breast cancer. Semin. Oncol..

[12] Carey LA (2006). Race, breast cancer subtypes, and survival in the Carolina breast cancer study. JAMA.

[13] Siddharth S, Sharma D (2018). Racial disparity and triple-negative breast cancer in African-American women: A multifaceted affair between obesity, biology, and socioeconomic determinants. Cancers.

[14] de Jong VMT (2022). Prognostic value of stromal tumor-infiltrating lymphocytes in young, node-negative, triple-negative breast cancer patients who did not receive (neo) adjuvant systemic therapy. J. Clin. Oncol..

[15] Gross J, Lapiere CM (1962). Collagenolytic activity in amphibian tissues: A tissue culture assay. Proc. Natl. Acad. Sci. U. S. A..

[16] Visse R, Nagase H (2003). Matrix metalloproteinases and tissue inhibitors of metalloproteinases: Structure, function, and biochemistry. Circ. Res..

[17] Lohi J, Wilson CL, Roby JD, Parks WC (2001). Epilysin, a novel human matrix metalloproteinase (MMP-28) expressed in testis and keratinocytes and in response to injury. J. Biol. Chem..

[18] Sternlicht MD, Werb Z (2001). How matrix metalloproteinases regulate cell behavior. Annu. Rev. Cell Dev. Biol..

[19] Malemud CJ (2006). Matrix metalloproteinases (MMPs) in health and disease: An overview. Front. Biosci..

[20] Lemaître V, D'Armiento J (2006). Matrix metalloproteinases in development and disease. Birth Defects Res. C. Embryo. Today.

[21] Deryugina EI, Quigley JP (2015). Tumor angiogenesis: MMP-mediated induction of intravasation- and metastasis-sustaining neovasculature. Matrix. Biol..

[22] Peng B (2010). Meta-analysis of association between matrix metalloproteinases 2, 7 and 9 promoter polymorphisms and cancer risk. Mutagenesis.

[23] Peng B (2010). Polymorphisms in the promoter regions of matrix metalloproteinases 1 and 3 and cancer risk: A meta-analysis of 50 case-control studies. Mutagenesis.

[24] McColgan P, Sharma P (2009). Polymorphisms of matrix metalloproteinases 1, 2, 3 and 9 and susceptibility to lung, breast and colorectal cancer in over 30,000 subjects. Int. J. Cancer.

[25] Huang C (2022). Epidemiological evidence between variants in matrix metalloproteinases-2, -7, and -9 and cancer risk. Front. Oncol..

[26] Xu T, Zhang S, Qiu D, Li X, Fan Y (2020). Association between matrix metalloproteinase 9 polymorphisms and breast cancer risk: An updated meta-analysis and trial sequential analysis. Gene.

[27] Han M (2017). Associations of MMP-2 −1306 C/T and MMP-9 −1562 C/T polymorphisms with breast cancer risk among different populations: A meta-analysis. Genes Genom..

[28] Ou YX, Bi R (2020). Meta-analysis on the relationship between the SNP of MMP-2-1306 C>T and susceptibility to breast cancer. Eur. Rev. Med. Pharmacol. Sci..

[29] Ren F (2015). Overexpression of MMP family members functions as prognostic biomarker for breast cancer patients: A systematic review and meta-analysis. PLoS ONE.

[30] Song J, Su H, Zhou YY, Guo LL (2013). Prognostic value of matrix metalloproteinase 9 expression in breast cancer patients: A meta-analysis. Asian Pac. J. Cancer. Prev..

[31] Chen Y, Wang X, Chen G, Dong C, Zhang D (2015). The impact of matrix metalloproteinase 2 on prognosis and clinicopathology of breast cancer patients: A systematic meta-analysis. PLoS ONE.

[32] Sui J, Huang J, Zhang Y (2021). The MMP-1 gene rs1799750 polymorphism is associated with breast cancer risk. Genet. Test. Mol. Biomarkers.

[33] Liu D (2012). Association between polymorphisms in the promoter regions of matrix metalloproteinases (MMPs) and risk of cancer metastasis: A meta-analysis. PLoS ONE.

[34] Hill HA (1994). A longitudinal analysis of predictors of quitting smoking among participants in a self-help intervention trial. Addict. Behav..

[35] Lee YH, Bae SC, Song GG (2013). Hepatitis B virus (HBV) reactivation in rheumatic patients with hepatitis core antigen (HBV occult carriers) undergoing anti-tumor necrosis factor therapy. Clin. Exp. Rheumatol..

[36] Chen YC (2019). Assessing causality between childhood adiposity and early puberty: A bidirectional mendelian randomization and longitudinal study. Metabolism.

[37] Bowden J, Holmes MV (2019). Meta-analysis and mendelian randomization: A review. Res Synth Methods.

[38] Burgess S, Small DS, Thompson SG (2017). A review of instrumental variable estimators for mendelian randomization. Stat. Methods. Med. Res..

[39] Emdin CA, Khera AV, Kathiresan S (2017). Mendelian randomization. JAMA.

[40] Yang Z, Yu R, Deng W, Wang W (2021). Genetic evidence for the causal association between programmed death-ligand 1 and lung cancer. J Cancer Res. Clin. Oncol..

[41] Chen D (2022). Assessing causality between osteoarthritis with urate levels and gout: A bidirectional Mendelian randomization study. Osteoarthr. Cartil..

[42] Davey Smith G, Hemani G (2014). Mendelian randomization: Genetic anchors for causal inference in epidemiological studies. Hum. Mol. Genet..

[43] Wang Q, Shi Q, Lu J, Wang Z, Hou J (2022). Causal relationships between inflammatory factors and multiple myeloma: A bidirectional Mendelian randomization study. Int. J. Cancer.

[44] Brinckerhoff CE, Rutter JL, Benbow U (2000). Interstitial collagenases as markers of tumor progression. Clin. Cancer. Res..

[45] Zhu Y (2022). Exosomal MMP-1 transfers metastasis potential in triple-negative breast cancer through PAR1-mediated EMT. Breast Cancer Res. Treat..

[46] Harati R, Hafezi S, Mabondzo A, Tlili A (2020). Silencing miR-202-3p increases MMP-1 and promotes a brain invasive phenotype in metastatic breast cancer cells. PLoS ONE.

[47] Wang QM, Lv L, Tang Y, Zhang L, Wang LF (2019). MMP-1 is overexpressed in triple-negative breast cancer tissues and the knockdown of MMP-1 expression inhibits tumor cell malignant behaviors in vitro. Oncol. Lett..

[48] Balkhi S, Mashayekhi F, Salehzadeh A, Saedi HS (2020). Matrix metalloproteinase (MMP)-1 and MMP-3 gene variations affect MMP-1 and -3 serum concentration and associates with breast cancer. Mol. Biol. Rep..

[49] Hughes S (2007). Matrix metalloproteinase single-nucleotide polymorphisms and haplotypes predict breast cancer progression. Clin. Cancer. Res..

[50] Padala C (2017). Synergistic effect of collagenase-1 (MMP1), stromelysin-1 (MMP3) and gelatinase-B (MMP9) gene polymorphisms in breast cancer. PLoS ONE.

[51] Boström P (2011). MMP-1 expression has an independent prognostic value in breast cancer. BMC Cancer.

[52] Hsiao CL (2018). The Association of matrix metalloproteinase-1 promoter polymorphisms with breast cancer. In Vivo.

[53] Białkowska K (2020). Polymorphisms in MMP-1, MMP-2, MMP-7, MMP-13 and MT2A do not contribute to breast, lung and colon cancer risk in polish population. Hered. Cancer Clin. Pract..

[54] Zhou P (2011). Current evidence on the relationship between four polymorphisms in the matrix metalloproteinases (MMP) gene and breast cancer risk: A meta-analysis. Breast Cancer Res. Treat..

[55] Kulić A, Dedić Plavetić N, Vrbanec J, Sirotković-Skerlev M (2012). Low serum MMP-1 in breast cancer: A negative prognostic factor?. Biomarkers.

[56] Kim GE (2014). Expression of matrix metalloproteinases and their inhibitors in different immunohistochemical-based molecular subtypes of breast cancer. BMC Cancer.

[57] Slattery ML (2013). Matrix metalloproteinase genes are associated with breast cancer risk and survival: The breast cancer health disparities study. PLoS ONE.

[58] Mirastschijski U (2019). Novel specific human and mouse stromelysin-1 (MMP-3) and stromelysin-2 (MMP-10) antibodies for biochemical and immunohistochemical analyses. Wound Repair Regen..

[59] Fang S (2005). Polymorphisms in the MMP1 and MMP3 promoter and non-small cell lung carcinoma in North China. Carcinogenesis.

[60] McMahan RS (2016). Stromelysin-2 (MMP10) moderates inflammation by controlling macrophage activation. J. Immunol..

[61] Piskór BM (2020). Plasma level of MMP-10 may be a prognostic marker in early stages of breast cancer. J. Clin. Med..

[62] Benson CS, Babu SD, Radhakrishna S, Selvamurugan N, Ravi Sankar B (2013). Expression of matrix metalloproteinases in human breast cancer tissues. Dis. Markers..

[63] Mehner C (2015). Tumor cell expression of MMP3 as a prognostic factor for poor survival in pancreatic, pulmonary, and mammary carcinoma. Genes. Cancer..

[64] Basu S, Thorat R, Dalal SN (2015). MMP7 is required to mediate cell invasion and tumor formation upon Plakophilin3 loss. PLoS ONE.

[65] Paulsson M (1992). Basement membrane proteins: Structure, assembly, and cellular interactions. Crit. Rev. Biochem. Mol. Biol..

[66] Chang J, Chaudhuri O (2019). Beyond proteases: Basement membrane mechanics and cancer in vasion. J. Cell. Biol..

[67] Piskór BM (2021). Plasma concentrations of Matrilysins MMP-7 and MMP-26 as diagnostic biomarkers in breast cancer. J. Clin. Med..

[68] Voorzanger-Rousselot N (2006). Association of 12 serum biochemical markers of angiogenesis, tumour invasion and bone turnover with bone metastases from breast cancer: A crossectional and longitudinal evaluation. Br. J. Cancer..

[69] Cao PL (2014). Expressions of FOXC1 and MMP-7 in molecular subtypes of breast cancer and their association with clinicopathological characteristics. Zhejiang Da Xue Xue Bao Yi Xue Ban.

[70] Katunina AI (2011). Matrix metalloproteinases 2, 7, and 9 in tumors and sera of patients with breast cancer. Bull. Exp. Biol. Med..

[71] Lawlor DA, Harbord RM, Sterne JA, Timpson N, Davey Smith G (2008). Mendelian randomization: Using genes as instruments for making causal inferences in epidemiology. Stat. Med..

[72] Ben Elsworth ML, Alexander T, Liu Y, Matthews P, Hallett J, Bates P, Palmer T, Haberland V, Smith GD, Zheng J, Haycock P, Gaunt TR, Hemani G (2020). The MRC IEU OpenGWAS data infrastructure. bioRxiv.

[73] Hemani G, Zheng J, Elsworth B, Wade KH, Baird D, Haberland V, Laurin C, Burgess S, Bowden J, Langdon R, Tan VY, Yarmolinsky J, Shihab HA, Timpson NJ, Evans DM, Relton C, Martin RM, Davey Smith G, Gaunt TR, Haycock PC (2018). The MR-Base platform supports systematic causal inference across the human phenome. Elife.

[74] Folkersen L (2020). Genomic and drug target evaluation of 90 cardiovascular proteins in 30,931 individuals. Nat. Metab..

[75] Staley JR (2016). PhenoScanner: A database of human genotype-phenotype associations. Bioinformatics.

[76] Kamat MA (2019). PhenoScanner V2: An expanded tool for searching human genotype-phenotype associations. Bioinformatics.

[77] Michailidou K (2017). Association analysis identifies 65 new breast cancer risk loci. Nature.

[78] Michailidou K (2015). Genome-wide association analysis of more than 120,000 individuals identifies 15 new susceptibility loci for breast cancer. Nat. Genet..

[79] Palmer TM (2012). Using multiple genetic variants as instrumental variables for modifiable risk factors. Stat. Methods. Med. Res..

[80] D., F. Introduction to quantitative genetics. Prentice. Hall. (1996).

[81] Bowden J (2017). A framework for the investigation of pleiotropy in two-sample summary data Mendelian randomization. Stat. Med..

[82] Burgess S, Bowden J, Fall T, Ingelsson E, Thompson SG (2017). Sensitivity analyses for robust causal inference from mendelian randomization analyses with multiple genetic variants. Epidemiology.

[83] Bowden J, Davey Smith G, Haycock PC, Burgess S (2016). Consistent estimation in mendelian randomization with some invalid instruments using a weighted median estimator. Genet. Epidemiol..

[84] Burgess S, Thompson SG (2017). Interpreting findings from mendelian randomization using the MR-Egger method. Eur. J. Epidemiol..

[85] Bowden J, Davey Smith G, Burgess S (2015). Mendelian randomization with invalid instruments: Effect estimation and bias detection through Egger regression. Int. J. Epidemiol..

[86] Verbanck M, Chen CY, Neale B, Do R (2018). Detection of widespread horizontal pleiotropy in causal relationships inferred from Mendelian randomization between complex traits and diseases. Nat. Genet..

[87] Bowden J (2016). Assessing the suitability of summary data for two-sample Mendelian randomization analyses using MR-Egger regression: The role of the I2 statistic. Int. J. Epidemiol..

