# Supplementary material for: Causal relationships between serum matrix metalloproteinases and estrogen receptor-negative breast cancer: a bidirectional mendelian randomization study
Source: Sci Rep. 2023 May 15;13:7849. doi: 10.1038/s41598-023-34200-0 (PMC10185521; doi:10.1038/s41598-023-34200-0)
Supplement: Supplementary file 1 — Supplementary Information. [file 41598_2023_34200_MOESM1_ESM.docx]

**Causal relationships between serum matrix metalloproteinases and estrogen receptor-negative breast cancer: A Bidirectional Mendelian Randomization Study**

**Zijun Zhao^1#^ Qing Cao^1#^ Ming Zhu ^1^ Chaonan Wang^1^ Xin Lu^1^**

1 Department of Surgery, Peking Union Medical College Hospital, Chinese Academy of Medical Sciences and Peking Union Medical College, Beijing, China.

Corresponding author: Xin Lu, MD, Professor, Department of Surgery, Peking Union Medical College Hospital, Chinese Academy of Medical Sciences and Peking Union Medical College, 1 Shuaifuyuan, Wangfujing, Beijing 100730, China. [luxin@pumch.cn](mailto:luxin@pumch.cn)

# ZJZ and QC contributed equally to this work

Email address:

Zijun Zhao: [fallingflower@163.com](mailto:fallingflower@163.com)

Qing Cao: [caoqingdoctor@163.com](mailto:caoqingdoctor@163.com)

Ming Zhu: [josephzhu08@126.com](mailto:josephzhu08@126.com)

Chaonan Wang: [dolly0215@sina.com](mailto:dolly0215@sina.com)

Xin Lu: [luxin@pumch.cn](mailto:luxin@pumch.cn)

**Supplementary Table S1. R^2^ and F-statistics for instrumental variables of MMP-1**

| **SNP** | **Beta of exposure** | **Eaf of exposure** | **SE of exposure** | **Sample size of exposure** | **R^2^** | **F** |
| --- | --- | --- | --- | --- | --- | --- |
| rs111839693 | -0.3441 | 0.0275 | 0.0398 | 21758 | 0.006333 | 138.6628 |
| rs117210823 | 0.2989 | 0.0239 | 0.0437 | 21758 | 0.004168 | 91.0683 |
| rs12141791 | 0.0829 | 0.6953 | 0.0134 | 21758 | 0.002912 | 63.53738 |
| rs141699001 | 0.2997 | 0.0206 | 0.0494 | 21758 | 0.003624 | 79.13831 |
| rs142336874 | -0.2063 | 0.0287 | 0.0365 | 21758 | 0.002373 | 51.74573 |
| rs146034621 | 0.3597 | 0.0512 | 0.0472 | 21758 | 0.012571 | 276.9673 |
| rs149498412 | -0.6855 | 0.0152 | 0.0692 | 21758 | 0.014068 | 310.4336 |
| rs150077704 | 0.2641 | 0.0222 | 0.0466 | 21758 | 0.003028 | 66.07938 |
| rs17860950 | 0.226 | 0.0663 | 0.024 | 21758 | 0.006324 | 138.4528 |
| rs180993745 | 0.5623 | 0.0121 | 0.0638 | 21758 | 0.007559 | 165.7062 |
| rs471994 | -0.3575 | 0.3679 | 0.0111 | 21758 | 0.059443 | 1374.964 |
| rs4754892 | 0.2557 | 0.0308 | 0.0389 | 21758 | 0.003904 | 85.25762 |
| rs531335 | -0.2744 | 0.9395 | 0.0241 | 21758 | 0.00856 | 187.8291 |
| rs669969 | 0.1817 | 0.9199 | 0.021 | 21758 | 0.004865 | 106.3678 |
| rs6993770 | -0.1235 | 0.2681 | 0.0125 | 21758 | 0.005986 | 131.0083 |
| rs7123658 | -0.1667 | 0.5712 | 0.0111 | 21758 | 0.013613 | 300.245 |

Eaf, Effect allele frequency; MMP, Matrix metalloproteinases; SE, standard error; SNP, single nucleotide polymorphisms

**Supplementary Table S2. R^2^ and F-statistics for instrumental variables of MMP-3**

| **SNP** | **Beta of exposure** | **Eaf of exposure** | **SE of exposure** | **Sample size of exposure** | **R^2^** | **F** |
| --- | --- | --- | --- | --- | --- | --- |
| rs11225455 | 0.1434 | 0.5272 | 0.0098 | 21758 | 0.010251 | 225.3384 |
| rs11606787 | -0.1205 | 0.3819 | 0.01 | 21758 | 0.006855 | 150.1685 |
| rs11668189 | -0.0557 | 0.3586 | 0.01 | 21758 | 0.001427 | 31.09417 |
| rs117470978 | 0.2078 | 0.0516 | 0.0262 | 21758 | 0.004226 | 92.33806 |
| rs117588360 | -0.2267 | 0.0253 | 0.0415 | 21758 | 0.002535 | 55.2848 |
| rs140637416 | 0.2192 | 0.0477 | 0.0191 | 21758 | 0.004365 | 95.38547 |
| rs146605342 | -0.4616 | 0.0179 | 0.0477 | 21758 | 0.007492 | 164.2159 |
| rs3020919 | 0.2572 | 0.2409 | 0.0106 | 21758 | 0.024194 | 539.4153 |
| rs35089914 | -0.1152 | 0.1171 | 0.014 | 21758 | 0.002744 | 59.86539 |
| rs471994 | 0.3597 | 0.3582 | 0.0094 | 21758 | 0.059489 | 1376.104 |
| rs491152 | -0.1557 | 0.9445 | 0.0236 | 21758 | 0.002542 | 55.4353 |
| rs7478816 | -0.1506 | 0.0876 | 0.0155 | 21758 | 0.003626 | 79.16364 |

Eaf, Effect allele frequency; MMP, Matrix metalloproteinases; SE, standard error; SNP, single nucleotide polymorphisms

**Supplementary Table S3. R^2^ and F-statistics for instrumental variables of MMP-7**

| **SNP** | **Beta of exposure** | **Eaf of exposure** | **SE of exposure** | **Sample size of exposure** | **R^2^** | **F** |
| --- | --- | --- | --- | --- | --- | --- |
| rs10791573 | -0.0707 | 0.2743 | 0.0122 | 21758 | 0.00199 | 43.38064 |
| rs1144393 | -0.0681 | 0.3713 | 0.0119 | 21758 | 0.002165 | 47.20772 |
| rs11568819 | 0.5584 | 0.0625 | 0.0244 | 21758 | 0.03654 | 825.1209 |
| rs117669898 | 0.2337 | 0.023 | 0.0414 | 21758 | 0.002455 | 53.53233 |
| rs118181466 | 0.218 | 0.0326 | 0.0357 | 21758 | 0.002998 | 65.4108 |
| rs12796179 | -0.123 | 0.2249 | 0.0138 | 21758 | 0.005275 | 115.3621 |
| rs1711431 | -0.1093 | 0.6897 | 0.0127 | 21758 | 0.005113 | 111.8196 |
| rs62133135 | -0.0652 | 0.3853 | 0.0114 | 21758 | 0.002014 | 43.89771 |
| rs9427716 | 0.0866 | 0.4615 | 0.0123 | 21758 | 0.003728 | 81.39995 |

Eaf, Effect allele frequency; MMP, Matrix metalloproteinases; SE, standard error; SNP, single nucleotide polymorphisms

**Supplementary Table S4. R^2^ and F-statistics for instrumental variables of MMP-10**

| **SNP** | **Beta of exposure** | **Eaf of exposure** | **SE of exposure** | **Sample size of exposure** | **R^2^** | **F** |
| --- | --- | --- | --- | --- | --- | --- |
| rs116936690 | 0.3269 | 0.0637 | 0.0238 | 21758 | 0.012747 | 280.9086 |
| rs17359230 | 0.2813 | 0.0332 | 0.0362 | 21758 | 0.00508 | 111.0798 |
| rs17860955 | -1.1276 | 0.0168 | 0.0443 | 21758 | 0.042004 | 953.9084 |
| rs1939533 | 0.123 | 0.1378 | 0.0167 | 21758 | 0.003595 | 78.49475 |
| rs28829934 | 0.0788 | 0.3951 | 0.012 | 21758 | 0.002968 | 64.7654 |
| rs2897917 | 0.0932 | 0.2366 | 0.0144 | 21758 | 0.003138 | 68.4814 |
| rs4291645 | -0.1462 | 0.1426 | 0.0185 | 21758 | 0.005227 | 114.3096 |
| rs486055 | -0.3437 | 0.1583 | 0.0154 | 21758 | 0.031479 | 707.1272 |
| rs657043 | 0.3635 | 0.0465 | 0.0318 | 21758 | 0.011717 | 257.9349 |

Eaf, Effect allele frequency; MMP, Matrix metalloproteinases; SE, standard error; SNP, single nucleotide polymorphisms

**Supplementary Table S5. R^2^ and F-statistics for instrumental variables of MMP-12**

| **SNP** | **Beta of exposure** | **Eaf of exposure** | **SE of exposure** | **Sample size of exposure** | **R^2^** | **F** |
| --- | --- | --- | --- | --- | --- | --- |
| rs1048250 | 0.1272 | 0.0648 | 0.0225 | 21758 | 0.001961028 | 42.74794797 |
| rs111559668 | -0.2434 | 0.044 | 0.0271 | 21758 | 0.004984042 | 108.9759632 |
| rs11225471 | 0.2837 | 0.1784 | 0.0139 | 21758 | 0.023594129 | 525.7177202 |
| rs117365877 | -0.215 | 0.0442 | 0.0279 | 21758 | 0.003905676 | 85.30506059 |
| rs117953762 | 0.2541 | 0.0192 | 0.0461 | 21758 | 0.002431762 | 53.03437421 |
| rs1201821 | -0.0947 | 0.1428 | 0.0173 | 21758 | 0.002195535 | 47.87115771 |
| rs12975366 | -0.0804 | 0.3984 | 0.0106 | 21758 | 0.003098627 | 67.62326044 |
| rs138856825 | -0.3563 | 0.0293 | 0.0358 | 21758 | 0.007221282 | 158.2489662 |
| rs141887775 | -0.2368 | 0.0896 | 0.0186 | 21758 | 0.009148158 | 200.8648661 |
| rs148292565 | 0.3384 | 0.0161 | 0.0524 | 21758 | 0.003628002 | 79.2182196 |
| rs17860949 | -0.1541 | 0.1229 | 0.0161 | 21758 | 0.005119603 | 111.9552447 |
| rs626750 | -0.4744 | 0.1898 | 0.0127 | 21758 | 0.069216208 | 1617.84921 |
| rs72980300 | -0.2317 | 0.0363 | 0.0311 | 21758 | 0.003756043 | 82.02455771 |
| rs78425657 | -0.2743 | 0.0328 | 0.0342 | 21758 | 0.004773883 | 104.3587883 |

Eaf, Effect allele frequency; MMP, Matrix metalloproteinases; SE, standard error; SNP, single nucleotide polymorphisms

**Supplementary Table S6. R^2^ and F-statistics for instrumental variables of ER-negative BC (ieu-a-1128) on MMP-1**

| **SNP** | **Beta of exposure** | **Eaf of exposure** | **Sample size of exposure** | **SE of exposure** | **R^2^** | **F** |
| --- | --- | --- | --- | --- | --- | --- |
| rs10069690 | 0.1613 | 0.2601 | 127442 | 0.013 | 0.010014 | 1289.107 |
| rs10096351 | 0.07 | 0.5455 | 127442 | 0.0114 | 0.00243 | 310.3966 |
| rs10179592 | 0.1156 | 0.8991 | 127442 | 0.0193 | 0.002425 | 309.7454 |
| rs10209121 | -0.066 | 0.56 | 127442 | 0.0113 | 0.002147 | 274.1559 |
| rs10885405 | 0.0716 | 0.4613 | 127442 | 0.0113 | 0.002548 | 325.5369 |
| rs10995201 | -0.0963 | 0.1582 | 127442 | 0.0159 | 0.00247 | 315.557 |
| rs11076805 | -0.0782 | 0.2543 | 127442 | 0.014 | 0.002319 | 296.2567 |
| rs11571833 | 0.4346 | 0.0081 | 127442 | 0.0588 | 0.003035 | 387.9611 |
| rs12129456 | -0.0835 | 0.4095 | 127442 | 0.0118 | 0.003372 | 431.1709 |
| rs12472404 | -0.0879 | 0.2316 | 127442 | 0.0137 | 0.00275 | 351.4272 |
| rs12870942 | 0.071 | 0.3176 | 127442 | 0.012 | 0.002185 | 279.0757 |
| rs12990503 | -0.0734 | 0.7368 | 127442 | 0.0126 | 0.00209 | 266.8528 |
| rs1432679 | -0.073 | 0.5656 | 127442 | 0.0113 | 0.002619 | 334.595 |
| rs17828955 | -0.0797 | 0.2649 | 127442 | 0.013 | 0.002474 | 316.0505 |
| rs183438976 | 0.1285 | 0.0997 | 127442 | 0.0186 | 0.002964 | 378.8905 |
| rs189268208 | 0.1116 | 0.0858 | 127442 | 0.0196 | 0.001954 | 249.4836 |
| rs2008198 | 0.0671 | 0.364 | 127442 | 0.0118 | 0.002085 | 266.223 |
| rs2169137 | -0.128 | 0.7396 | 127442 | 0.0128 | 0.006311 | 809.3624 |
| rs2263146 | 0.0797 | 0.3504 | 127442 | 0.0118 | 0.002892 | 369.59 |
| rs2731830 | -0.2345 | 0.9644 | 127442 | 0.0419 | 0.003776 | 483.0272 |
| rs2747652 | 0.0991 | 0.526 | 127442 | 0.0113 | 0.004897 | 627.1612 |
| rs34810249 | 0.0804 | 0.2438 | 127442 | 0.0138 | 0.002383 | 304.4771 |
| rs3769823 | -0.0684 | 0.7071 | 127442 | 0.0121 | 0.001938 | 247.4518 |
| rs4143044 | 0.0833 | 0.7661 | 127442 | 0.0135 | 0.002487 | 317.7041 |
| rs4528762 | -0.1213 | 0.2646 | 127442 | 0.0147 | 0.005726 | 733.9472 |
| rs4754322 | -0.0629 | 0.4157 | 127442 | 0.0115 | 0.001922 | 245.4079 |
| rs4784227 | 0.1368 | 0.2403 | 127442 | 0.0128 | 0.006833 | 876.7615 |
| rs55872725 | -0.0784 | 0.4152 | 127442 | 0.0115 | 0.002985 | 381.5319 |
| rs56069439 | 0.155 | 0.2971 | 127442 | 0.0121 | 0.010034 | 1291.74 |
| rs56309329 | 0.0734 | 0.2368 | 127442 | 0.0131 | 0.001947 | 248.6534 |
| rs56687477 | -0.1114 | 0.1577 | 127442 | 0.016 | 0.003297 | 421.54 |
| rs616402 | -0.1172 | 0.307 | 127442 | 0.0124 | 0.005845 | 749.2182 |
| rs6569648 | 0.0741 | 0.7651 | 127442 | 0.0135 | 0.001974 | 252.0179 |
| rs6725517 | -0.0683 | 0.4065 | 127442 | 0.0122 | 0.002251 | 287.4995 |
| rs76956704 | 0.2193 | 0.03 | 127442 | 0.0325 | 0.002799 | 357.7036 |
| rs7710996 | -0.0773 | 0.7729 | 127442 | 0.0132 | 0.002098 | 267.8842 |
| rs9397437 | 0.2733 | 0.0682 | 127442 | 0.0206 | 0.009493 | 1221.419 |

Eaf, Effect allele frequency; ER-negative BC, estrogen receptor-negative breast cancer; MMP, Matrix metalloproteinases; SE, standard error; SNP, single nucleotide polymorphisms

**Supplementary Table S7. R^2^ and F-statistics for instrumental variables of ER-negative BC (ieu-a-1135) on MMP-1**

| **SNP** | **Beta of exposure** | **Eaf of exposure** | **Sample size of exposure** | **SE of exposure** | **R^2^** | **F** |
| --- | --- | --- | --- | --- | --- | --- |
| rs10069690 | 0.16997 | 0.258595 | 55149 | 0.01801 | 0.011078 | 617.7459 |
| rs10096351 | 0.0927 | 0.543728 | 55149 | 0.01645 | 0.004264 | 236.1416 |
| rs11571815 | 0.45564 | 0.0079461 | 55149 | 0.07826 | 0.003273 | 181.0959 |
| rs2169137 | -0.11944 | 0.739625 | 55149 | 0.01811 | 0.005495 | 304.6879 |
| rs4255510 | -0.09374 | 0.420466 | 55149 | 0.01661 | 0.004282 | 237.1786 |
| rs4528762 | -0.1174 | 0.265796 | 55149 | 0.01946 | 0.005379 | 298.2605 |
| rs4784227 | 0.13483 | 0.240383 | 55149 | 0.01846 | 0.006639 | 368.5668 |
| rs4849879 | 0.1533 | 0.879665 | 55149 | 0.02598 | 0.004975 | 275.7475 |
| rs58847541 | 0.12292 | 0.145241 | 55149 | 0.02244 | 0.003752 | 207.6645 |
| rs616402 | -0.12368 | 0.303524 | 55149 | 0.01799 | 0.006467 | 358.9781 |
| rs67397200 | 0.15918 | 0.295697 | 55149 | 0.01744 | 0.010554 | 588.2246 |
| rs72997345 | 0.44407 | 0.0118918 | 55149 | 0.07763 | 0.004634 | 256.7581 |
| rs74450259 | 0.24014 | 0.0390487 | 55149 | 0.0424 | 0.004328 | 239.7025 |
| rs9397437 | 0.27995 | 0.069152 | 55149 | 0.02917 | 0.01009 | 562.083 |

Eaf, Effect allele frequency; ER-negative BC, estrogen receptor-negative breast cancer; MMP, Matrix metalloproteinases; SE, standard error; SNP, single nucleotide polymorphisms

**Supplementary Table S8. R^2^ and F-statistics for instrumental variables of ER-negative BC (ieu-a-1136) on MMP-1**

| **SNP** | **Beta of exposure** | **Eaf of exposure** | **SE of exposure** | **Sample size of exposure** | **R^2^** | **F** |
| --- | --- | --- | --- | --- | --- | --- |
| rs10419397 | 0.15081 | 0.298018 | 0.02045 | 50225 | 0.009516 | 482.5185 |
| rs112149573 | 0.15238 | 0.23349 | 0.02155 | 50225 | 0.008311 | 420.9198 |
| rs113136187 | 0.19498 | 0.159887 | 0.02632 | 50225 | 0.010213 | 518.2293 |
| rs2731830 | -0.38679 | 0.961587 | 0.06797 | 50225 | 0.011052 | 561.2761 |
| rs4245739 | -0.14236 | 0.741452 | 0.02117 | 50225 | 0.00777 | 393.2969 |
| rs6913578 | 0.15469 | 0.307396 | 0.02027 | 50225 | 0.010189 | 516.9971 |
| rs910416 | 0.11959 | 0.529129 | 0.01916 | 50225 | 0.007127 | 360.489 |

Eaf, Effect allele frequency; ER-negative BC, estrogen receptor-negative breast cancer; MMP, Matrix metalloproteinases; SE, standard error; SNP, single nucleotide polymorphisms

**Supplementary Table S9. R^2^ and F-statistics for instrumental variables of ER-negative BC (ieu-a-1166) on MMP-1**

| **SNP** | **Beta of exposure** | **Eaf of exposure** | **SE of exposure** | **Sample size of exposure** | **R^2^** | **F** |
| --- | --- | --- | --- | --- | --- | --- |
| rs10419397 | 0.153133 | 0.297992 | 0.0202828 | 21695 | 0.009811 | 214.9393 |
| rs1421085 | -0.105432 | 0.41315 | 0.0192469 | 21695 | 0.00539 | 117.5646 |
| rs2125855 | -0.143924 | 0.742366 | 0.0210451 | 21695 | 0.007924 | 173.2575 |
| rs2242652 | 0.169725 | 0.205153 | 0.0229764 | 21695 | 0.009395 | 205.7321 |
| rs4784226 | 0.152251 | 0.233261 | 0.0213819 | 21695 | 0.008292 | 181.3742 |
| rs6913578 | 0.154671 | 0.306487 | 0.0201162 | 21695 | 0.01017 | 222.8812 |
| rs7297051 | -0.14107 | 0.241252 | 0.0227375 | 21695 | 0.007286 | 159.2074 |
| rs910416 | 0.121602 | 0.531864 | 0.0190191 | 21695 | 0.007363 | 160.9213 |

Eaf, Effect allele frequency; ER-negative BC, estrogen receptor-negative breast cancer; MMP, Matrix metalloproteinases; SE, standard error; SNP, single nucleotide polymorphisms


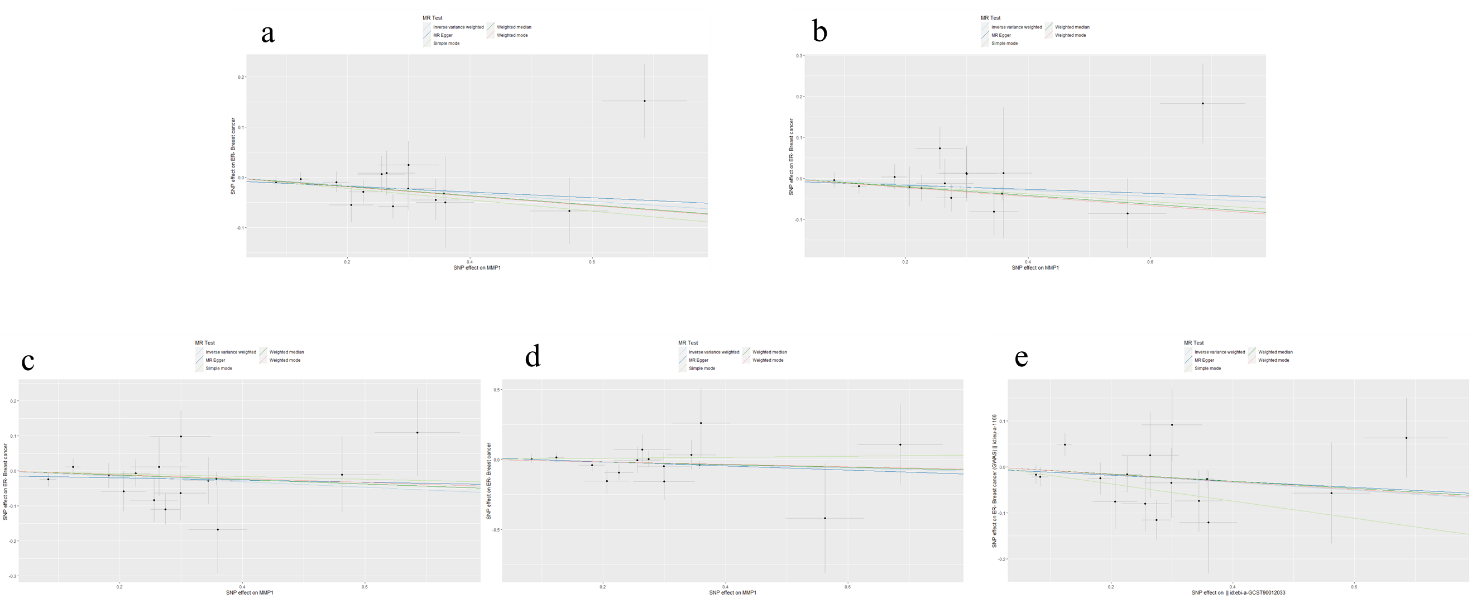


**Supplementary Figure S1. Scatter plots for analysis of causal effect of MMP-1 on ER-negative BC.**

(a) Associations between MMP-1 and ER-negative BC (experimental set: ieu-a-1128); (b) Associations between MMP-1 and ER-negative BC (Validation set 1: ieu-a-1135); (c) Associations between MMP-1 and ER-negative BC (Validation set 2: ieu-a-1136); (d) Associations between MMP-1 and ER-negative BC (Validation set 3: ieu-a-1137); (e) Associations between MMP-1 and ER-negative BC (Validation set 4: ieu-a-1166)

MMP, matrix metalloproteinases; ER-negative BC, estrogen receptor-negative breast cancer


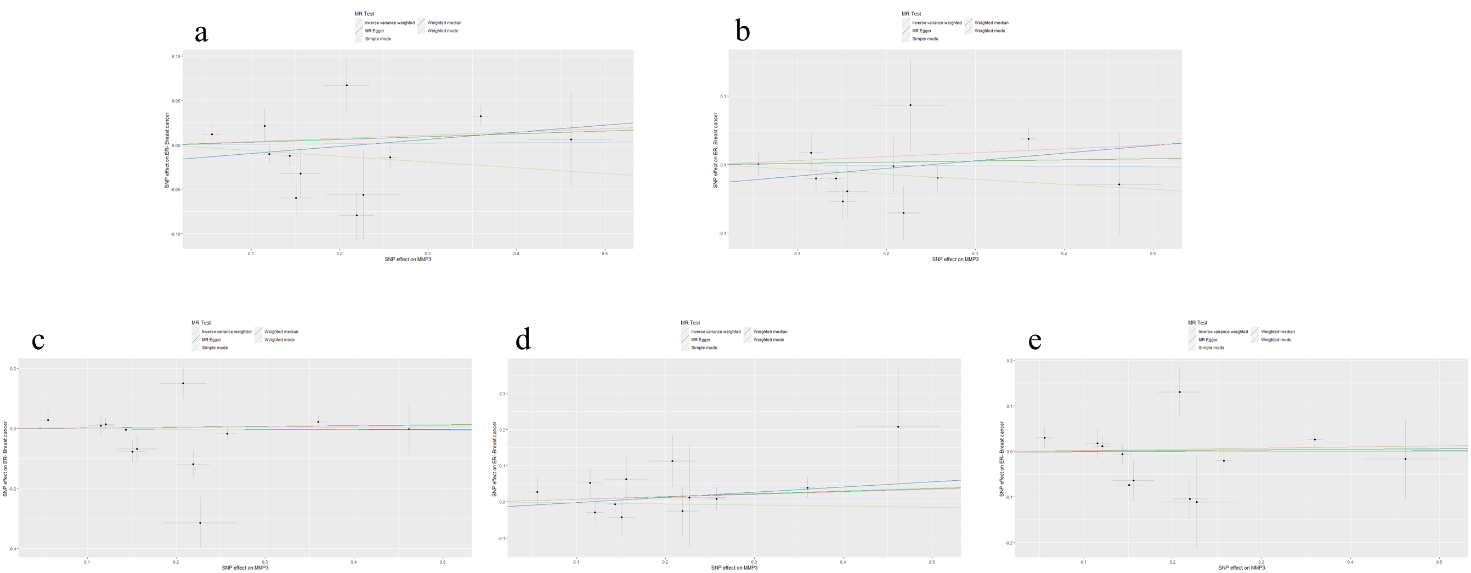


**Supplementary Figure S2. Scatter plots for analysis of causal effect of MMP-3 on ER-negative BC.**

(a) Associations between MMP-3 and ER-negative BC (experimental set: ieu-a-1128); (b) Associations between MMP-3 and ER-negative BC (Validation set 1: ieu-a-1135); (c) Associations between MMP-3 and ER-negative BC (Validation set 2: ieu-a-1136); (d) Associations between MMP-3 and ER-negative BC (Validation set 3: ieu-a-1137); (e) Associations between MMP-3 and ER-negative BC (Validation set 4: ieu-a-1166)

MMP, matrix metalloproteinases; ER-negative BC, estrogen receptor-negative breast cancer


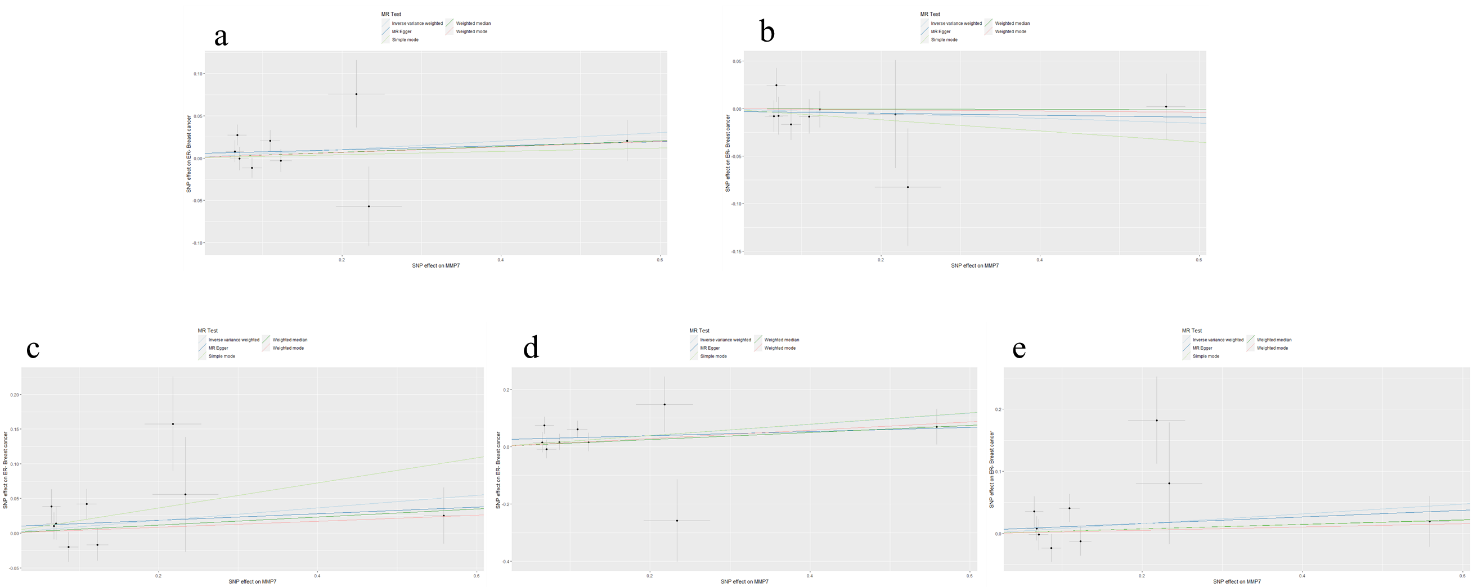


**Supplementary Figure S3. Scatter plots for analysis of causal effect of MMP-7 on ER-negative BC.**

(a) Associations between MMP-7 and ER-negative BC (experimental set: ieu-a-1128); (b) Associations between MMP-7 and ER-negative BC (Validation set 1: ieu-a-1135); (c) Associations between MMP-7 and ER-negative BC (Validation set 2: ieu-a-1136); (d) Associations between MMP-7 and ER-negative BC (Validation set 3: ieu-a-1137); (e) Associations between MMP-7 and ER-negative BC (Validation set 4: ieu-a-1166)

MMP, matrix metalloproteinases; ER-negative BC, estrogen receptor-negative breast cancer


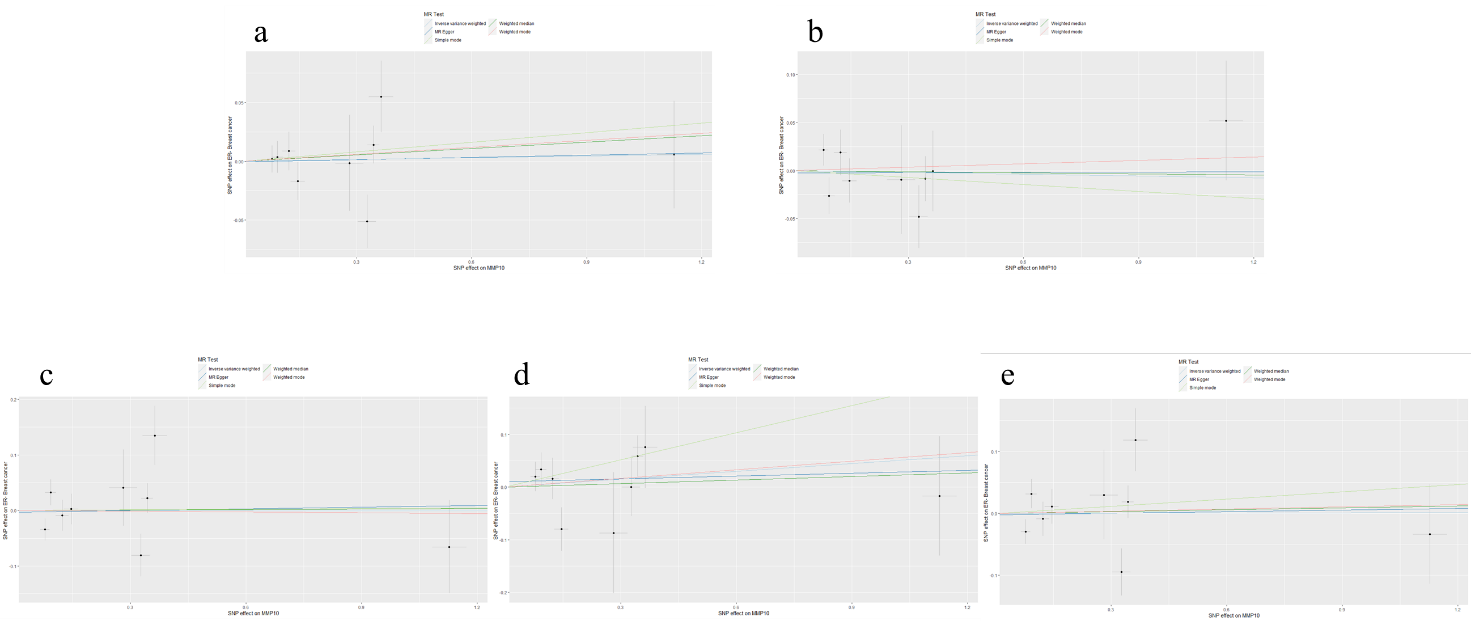


**Supplementary Figure S4. Scatter plots for analysis of causal effect of MMP-10 on ER-negative BC.**

(a) Associations between MMP-10 and ER-negative BC (experimental set: ieu-a-1128); (b) Associations between MMP-10 and ER-negative BC (Validation set 1: ieu-a-1135); (c) Associations between MMP-10 and ER-negative BC (Validation set 2: ieu-a-1136); (d) Associations between MMP-10 and ER-negative BC (Validation set 3: ieu-a-1137); (e) Associations between MMP-10 and ER-negative BC (Validation set 4: ieu-a-1166)

MMP, matrix metalloproteinases; ER-negative BC, estrogen receptor-negative breast cancer


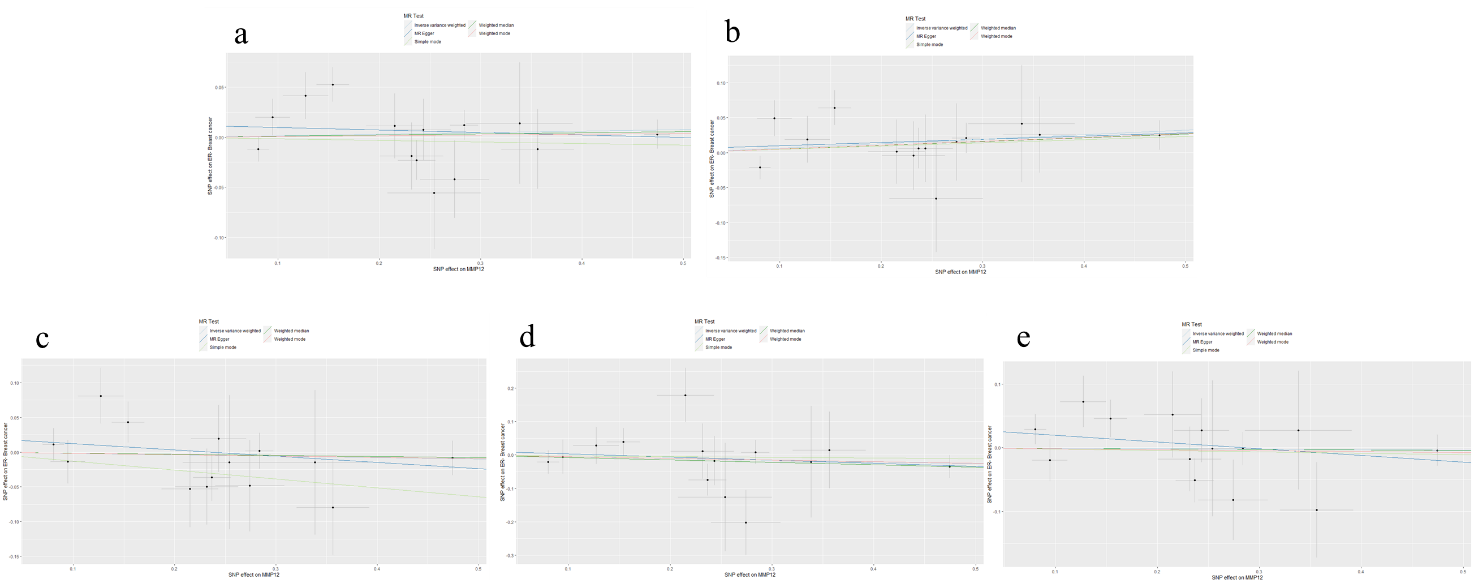


**Supplementary Figure S5. Scatter plots for analysis of causal effect of MMP-12 on ER-negative BC.**

(a) Associations between MMP-12 and ER-negative BC (experimental set: ieu-a-1128); (b) Associations between MMP-12 and ER-negative BC (Validation set 1: ieu-a-1135); (c) Associations between MMP-12 and ER-negative BC (Validation set 2: ieu-a-1136); (d) Associations between MMP-12 and ER-negative BC (Validation set 3: ieu-a-1137); (e) Associations between MMP-12 and ER-negative BC (Validation set 4: ieu-a-1166)

MMP, matrix metalloproteinases; ER-negative BC, estrogen receptor-negative breast cancer


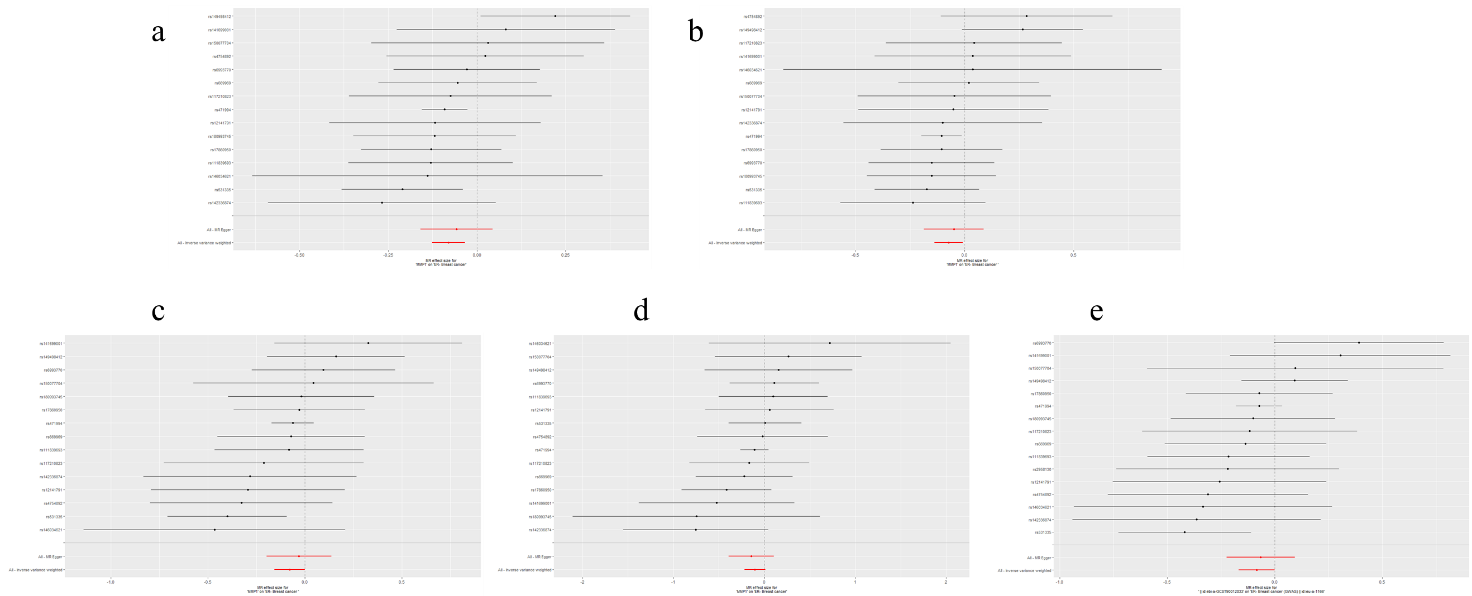


**Supplementary Figure S6. Forest plots for analysis of causal effect of MMP-1 on ER-negative BC.**

(a) Associations between MMP-1 and ER-negative BC (experimental set: ieu-a-1128); (b) Associations between MMP-1 and ER-negative BC (Validation set 1: ieu-a-1135); (c) Associations between MMP-1 and ER-negative BC (Validation set 2: ieu-a-1136); (d) Associations between MMP-1 and ER-negative BC (Validation set 3: ieu-a-1137); (e) Associations between MMP-1 and ER-negative BC (Validation set 4: ieu-a-1166)

MMP, matrix metalloproteinases; ER-negative BC, estrogen receptor-negative breast cancer


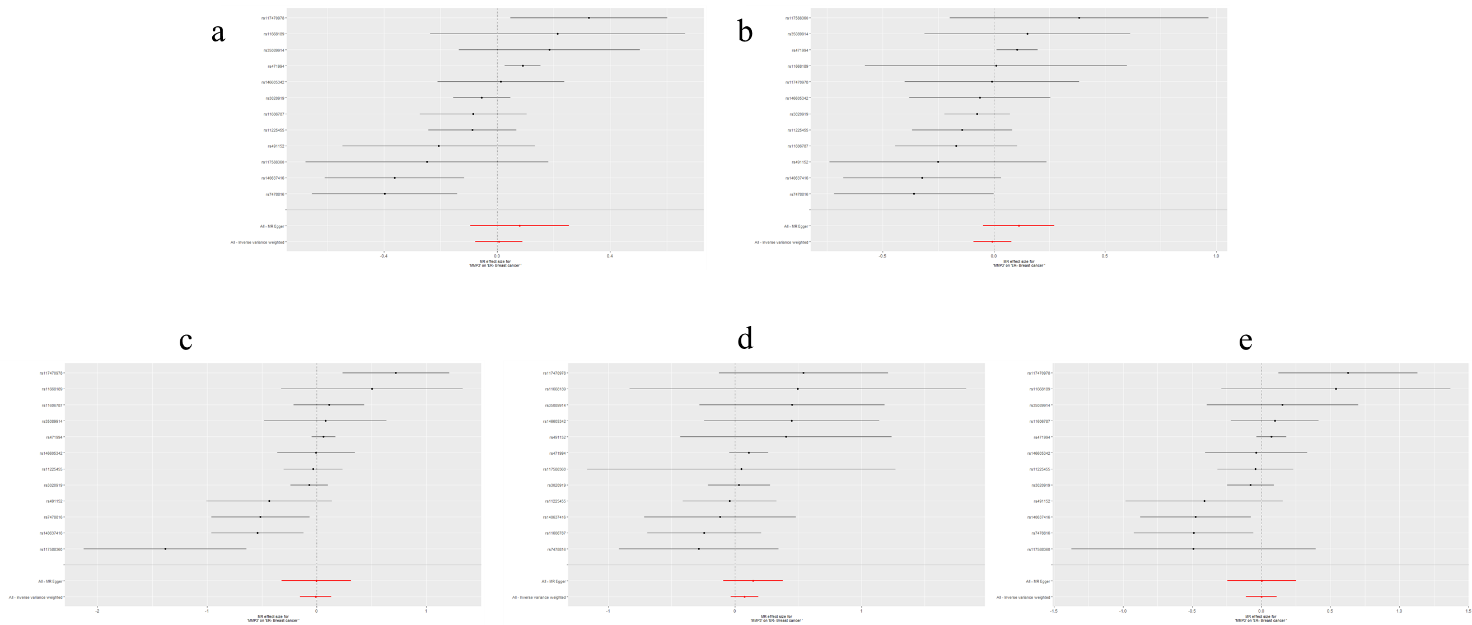


**Supplementary Figure S7. Forest plots for analysis of causal effect of MMP-3 on ER-negative BC.**

(a) Associations between MMP-3 and ER-negative BC (experimental set: ieu-a-1128); (b) Associations between MMP-3 and ER-negative BC (Validation set 1: ieu-a-1135); (c) Associations between MMP-3 and ER-negative BC (Validation set 2: ieu-a-1136); (d) Associations between MMP-3 and ER-negative BC (Validation set 3: ieu-a-1137); (e) Associations between MMP-3 and ER-negative BC (Validation set 4: ieu-a-1166)

MMP, matrix metalloproteinases; ER-negative BC, estrogen receptor-negative breast cancer


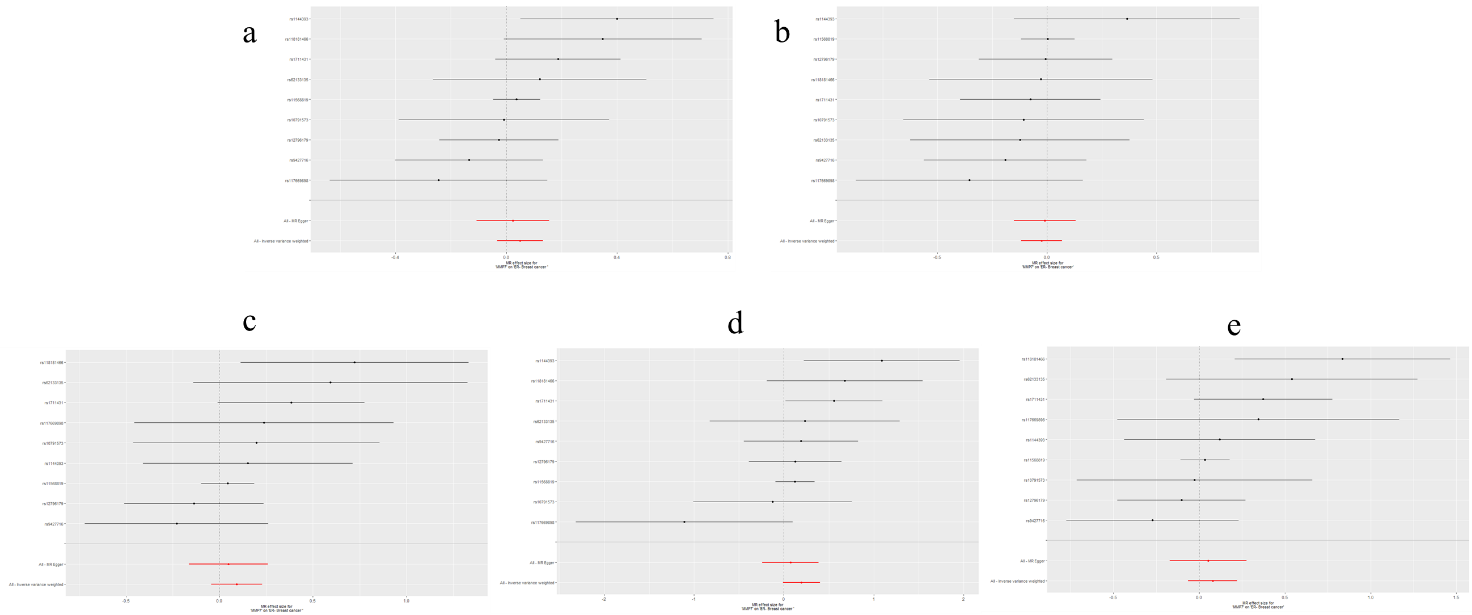


**Supplementary Figure S8. Forest plots for analysis of causal effect of MMP-7 on ER-negative BC.**

(a) Associations between MMP-7 and ER-negative BC (experimental set: ieu-a-1128); (b) Associations between MMP-7 and ER-negative BC (Validation set 1: ieu-a-1135); (c) Associations between MMP-7 and ER-negative BC (Validation set 2: ieu-a-1136); (d) Associations between MMP-7 and ER-negative BC (Validation set 3: ieu-a-1137); (e) Associations between MMP-7 and ER-negative BC (Validation set 4: ieu-a-1166)

MMP, matrix metalloproteinases; ER-negative BC, estrogen receptor-negative breast cancer


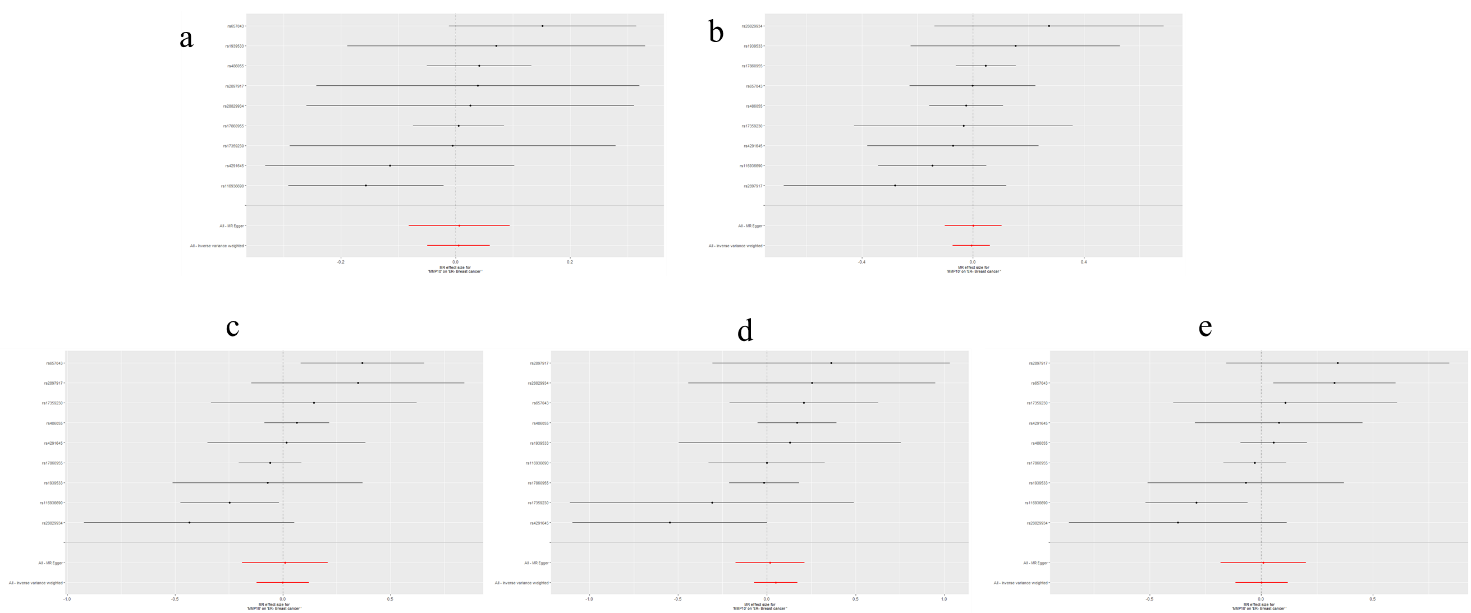


**Supplementary Figure S9. Forest plots for analysis of causal effect of MMP-10 on ER-negative BC.**

(a) Associations between MMP-10 and ER-negative BC (experimental set: ieu-a-1128); (b) Associations between MMP-10 and ER-negative BC (Validation set 1: ieu-a-1135); (c) Associations between MMP-10 and ER-negative BC (Validation set 2: ieu-a-1136); (d) Associations between MMP-10 and ER-negative BC (Validation set 3: ieu-a-1137); (e) Associations between MMP-10 and ER-negative BC (Validation set 4: ieu-a-1166)

MMP, matrix metalloproteinases; ER-negative BC, estrogen receptor-negative breast cancer


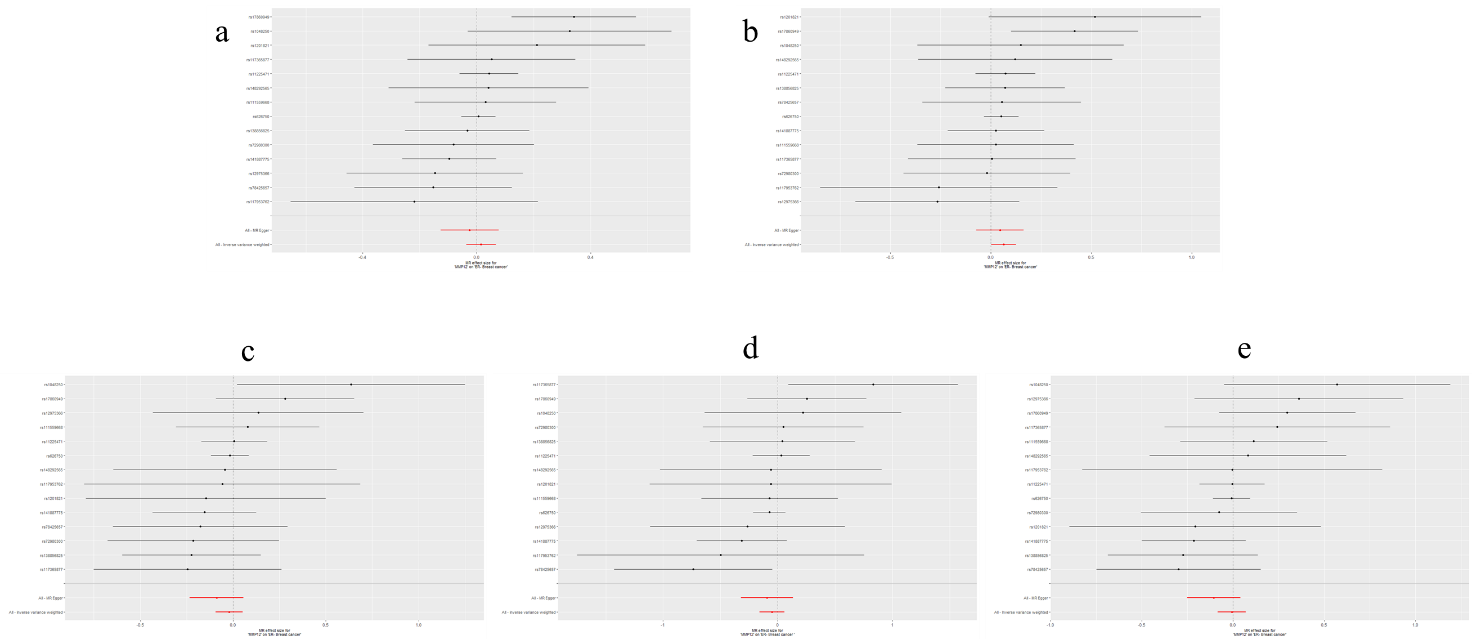


**Supplementary Figure S10. Forest plots for analysis of causal effect of MMP-12 on ER-negative BC.**

(a) Associations between MMP-12 and ER-negative BC (experimental set: ieu-a-1128); (b) Associations between MMP-12 and ER-negative BC (Validation set 1: ieu-a-1135); (c) Associations between MMP-12 and ER-negative BC (Validation set 2: ieu-a-1136); (d) Associations between MMP-12 and ER-negative BC (Validation set 3: ieu-a-1137); (e) Associations between MMP-12 and ER-negative BC (Validation set 4: ieu-a-1166)

MMP, matrix metalloproteinases; ER-negative BC, estrogen receptor-negative breast cancer


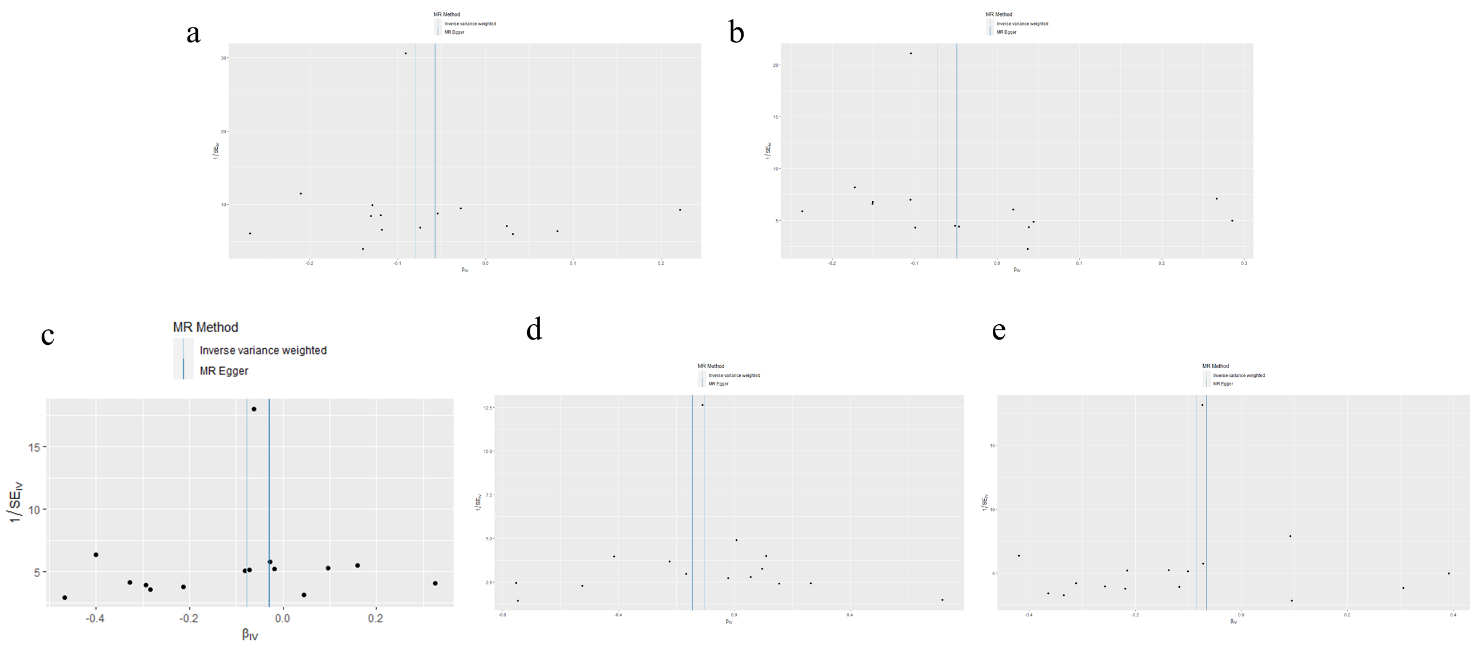


**Supplementary Figure S11. Funnel plots for analysis of causal effect of MMP-1 on ER-negative BC.**

(a) Associations between MMP-1 and ER-negative BC (experimental set: ieu-a-1128); (b) Associations between MMP-1 and ER-negative BC (Validation set 1: ieu-a-1135); (c) Associations between MMP-1 and ER-negative BC (Validation set 2: ieu-a-1136); (d) Associations between MMP-1 and ER-negative BC (Validation set 3: ieu-a-1137); (e) Associations between MMP-1 and ER-negative BC (Validation set 4: ieu-a-1166)

MMP, matrix metalloproteinases; ER-negative BC, estrogen receptor-negative breast cancer


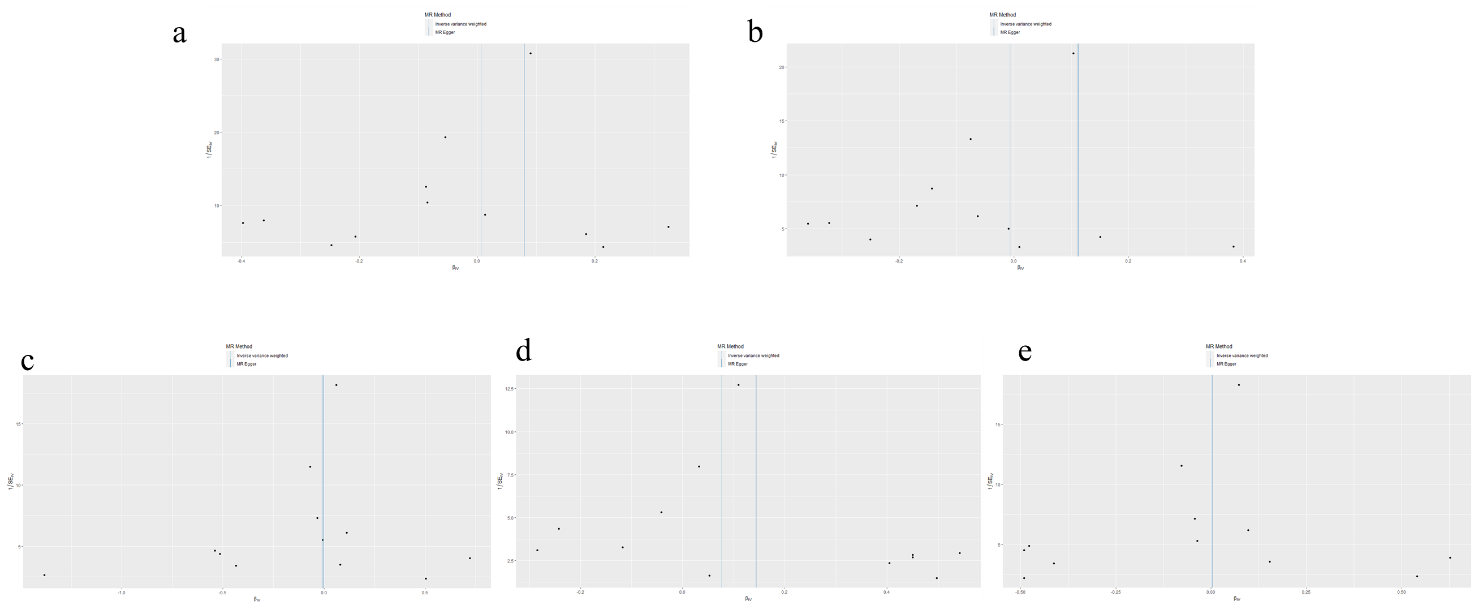


**Supplementary Figure S12. Funnel plots for analysis of causal effect of MMP-3 on ER-negative BC.**

(a) Associations between MMP-3 and ER-negative BC (experimental set: ieu-a-1128); (b) Associations between MMP-3 and ER-negative BC (Validation set 1: ieu-a-1135); (c) Associations between MMP-3 and ER-negative BC (Validation set 2: ieu-a-1136); (d) Associations between MMP-3 and ER-negative BC (Validation set 3: ieu-a-1137); (e) Associations between MMP-3 and ER-negative BC (Validation set 4: ieu-a-1166)

MMP, matrix metalloproteinases; ER-negative BC, estrogen receptor-negative breast cancer


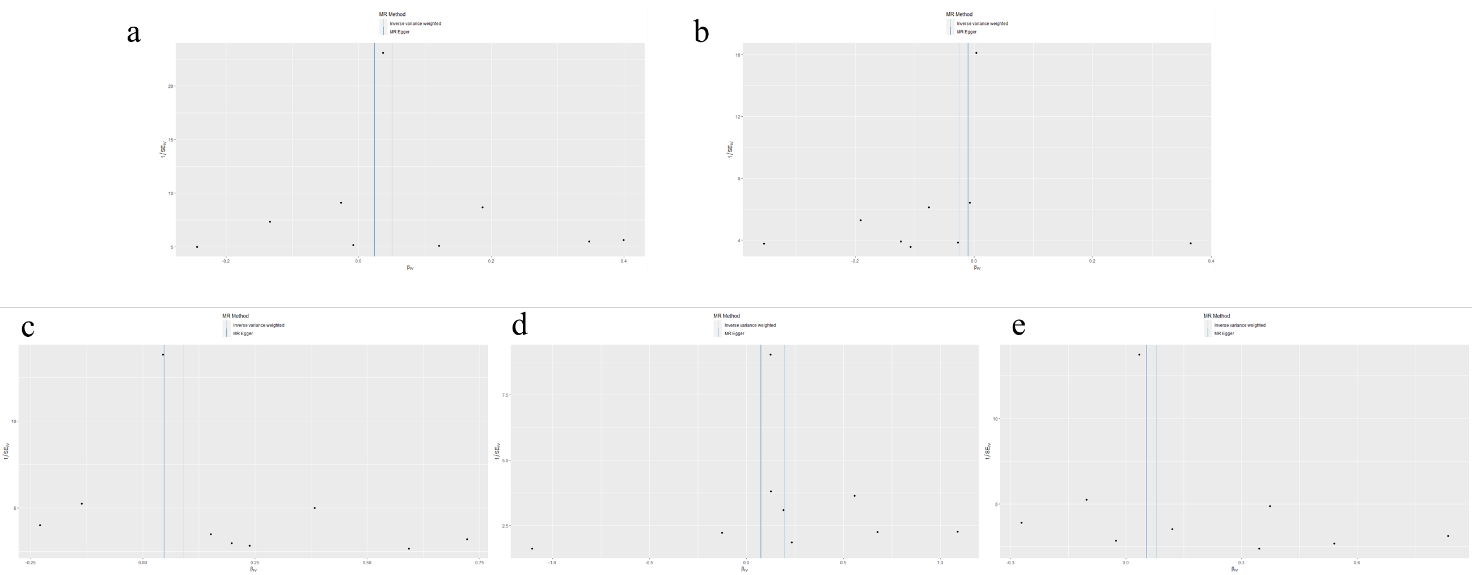


**Supplementary Figure S13. Funnel plots for analysis of causal effect of MMP-7 on ER-negative BC.**

(a) Associations between MMP-7 and ER-negative BC (experimental set: ieu-a-1128); (b) Associations between MMP-7 and ER-negative BC (Validation set 1: ieu-a-1135); (c) Associations between MMP-7 and ER-negative BC (Validation set 2: ieu-a-1136); (d) Associations between MMP-7 and ER-negative BC (Validation set 3: ieu-a-1137); (e) Associations between MMP-7 and ER-negative BC (Validation set 4: ieu-a-1166)

MMP, matrix metalloproteinases; ER-negative BC, estrogen receptor-negative breast cancer


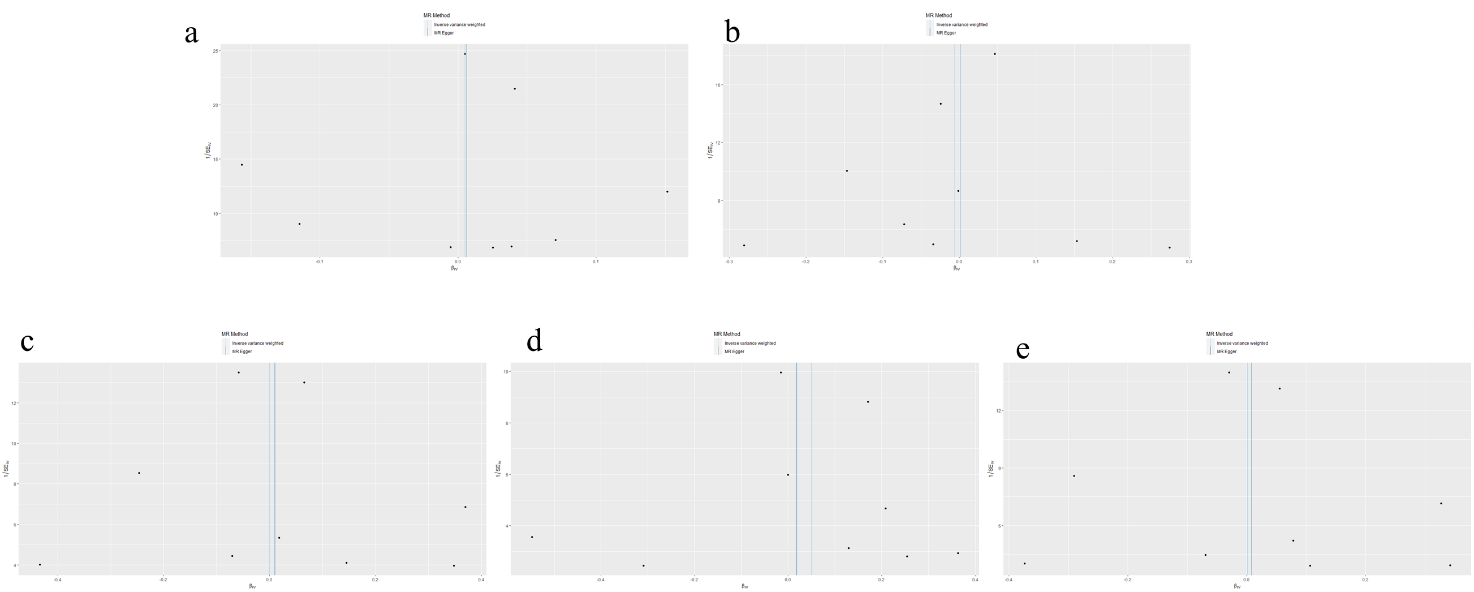


**Supplementary Figure S14. Funnel plots for analysis of causal effect of MMP-10 on ER-negative BC.**

(a) Associations between MMP-10 and ER-negative BC (experimental set: ieu-a-1128); (b) Associations between MMP-10 and ER-negative BC (Validation set 1: ieu-a-1135); (c) Associations between MMP-10 and ER-negative BC (Validation set 2: ieu-a-1136); (d) Associations between MMP-10 and ER-negative BC (Validation set 3: ieu-a-1137); (e) Associations between MMP-10 and ER-negative BC (Validation set 4: ieu-a-1166)

MMP, matrix metalloproteinases; ER-negative BC, estrogen receptor-negative breast cancer


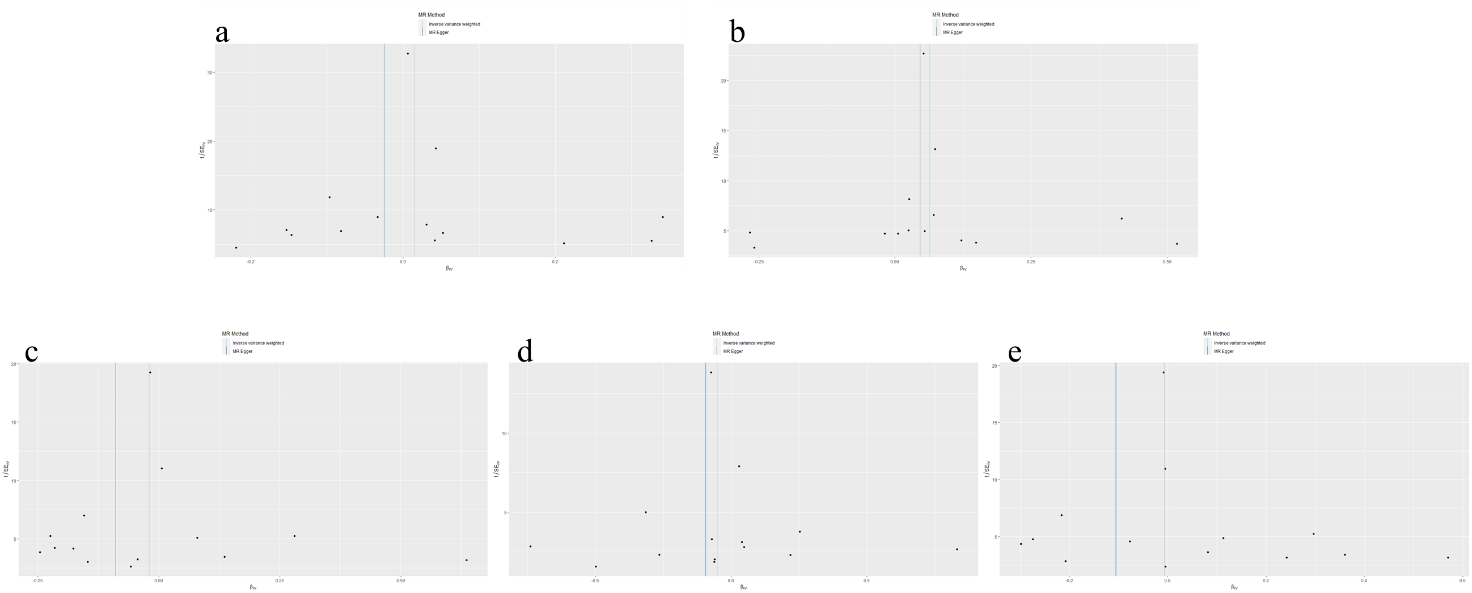


**Supplementary Figure S15. Funnel plots for analysis of causal effect of MMP-12 on ER-negative BC.**

(a) Associations between MMP-12 and ER-negative BC (experimental set: ieu-a-1128); (b) Associations between MMP-12 and ER-negative BC (Validation set 1: ieu-a-1135); (c) Associations between MMP-12 and ER-negative BC (Validation set 2: ieu-a-1136); (d) Associations between MMP-12 and ER-negative BC (Validation set 3: ieu-a-1137); (e) Associations between MMP-12 and ER-negative BC (Validation set 4: ieu-a-1166)

MMP, matrix metalloproteinases; ER-negative BC, estrogen receptor-negative breast cancer
